# ΔNp63 bookmarks and creates an accessible epigenetic environment for TGFβ-induced cancer cell stemness and invasiveness

**DOI:** 10.1186/s12964-024-01794-5

**Published:** 2024-08-23

**Authors:** Eleftheria Vasilaki, Yu Bai, Mohamad Moustafa Ali, Anders Sundqvist, Aristidis Moustakas, Carl-Henrik Heldin

**Affiliations:** 1 Department of Medical Biochemistry and Microbiology, Science for Life Laboratory, Uppsala University, Box 582, Uppsala, SE-751 23 Sweden; 2 Department of Immunology, Genetics and Pathology, Science for Life Laboratory, Uppsala University, Uppsala, SE-751 85 Sweden; 3 Department of Pharmaceutical Biosciences, Uppsala University, Box 591, Uppsala, SE-751 24 Sweden

**Keywords:** p63, Transforming growth factor β (TGFβ), Signal transduction, Transcription, Chromatin accessibility, Protein-protein interaction

## Abstract

**Background:**

p63 is a transcription factor with intrinsic pioneer factor activity and pleiotropic functions. Transforming growth factor β (TGFβ) signaling via activation and cooperative action of canonical, SMAD, and non-canonical, MAP-kinase (MAPK) pathways, elicits both anti- and pro-tumorigenic properties, including cell stemness and invasiveness. TGFβ activates the ΔNp63 transcriptional program in cancer cells; however, the link between TGFβ and p63 in unmasking the epigenetic landscape during tumor progression allowing chromatin accessibility and gene transcription, is not yet reported.

**Methods:**

Small molecule inhibitors, including protein kinase inhibitors and RNA-silencing, provided loss of function analyses. Sphere formation assays in cancer cells, chromatin immunoprecipitation and mRNA expression assays were utilized in order to gain mechanistic evidence. Mass spectrometry analysis coupled to co-immunoprecipitation assays revealed novel p63 interactors and their involvement in p63-dependent transcription.

**Results:**

The sphere-forming capacity of breast cancer cells was enhanced upon TGFβ stimulation and significantly decreased upon ΔNp63 depletion. Activation of TGFβ signaling via p38 MAPK signaling induced ΔNp63 phosphorylation at Ser 66/68 resulting in stabilized ΔNp63 protein with enhanced DNA binding properties. TGFβ stimulation altered the ratio of H3K27ac and H3K27me3 histone modification marks, pointing towards higher H3K27ac and increased p300 acetyltransferase recruitment to chromatin. By silencing the expression of ΔNp63, the TGFβ effect on chromatin remodeling was abrogated. Inhibition of H3K27me3, revealed the important role of TGFβ as the upstream signal for guiding ΔNp63 to the TGFβ/SMAD gene loci, as well as the indispensable role of ΔNp63 in recruiting histone modifying enzymes, such as p300, to these genomic regions, regulating chromatin accessibility and gene transcription. Mechanistically, TGFβ through SMAD activation induced dissociation of ΔNp63 from NURD or NCOR/SMRT histone deacetylation complexes, while promoted the assembly of ΔNp63-p300 complexes, affecting the levels of histone acetylation and the outcome of ΔNp63-dependent transcription.

**Conclusions:**

ΔNp63, phosphorylated and recruited by TGFβ to the TGFβ/SMAD/ΔNp63 gene loci, promotes chromatin accessibility and transcription of target genes related to stemness and cell invasion.

**Supplementary Information:**

The online version contains supplementary material available at 10.1186/s12964-024-01794-5.

## Background

p63 is a member of the p53 family of transcription factors. Mutations in the *TP63* gene cause human developmental defects, including limb deformation, cleft lip/palate, and ectodermal dysplasia [1]. p63 is known as the guardian of human reproduction, monitoring the integrity of the female germ line [2, 3]. In contrast to the high mutational rate of *TP53* in human cancers, *TP63* mutations are rare. Yet, recent studies implicate p63 in both anti- and pro-tumorigenic processes, including cell proliferation, differentiation, senescence, invasion and metastasis [4, 5].

p63’s pleiotropic functions are partly dependent on the differential and tissue-specific expression of multiple p63 isoforms derived from distinct promoters or alternative splicing at the end of the *TP63* gene. The TAp63 proteins contain an N-terminal transactivation (TA) domain, while ΔNp63 isoforms are transcribed from an alternative promoter and contain an activation domain composed of fourteen unique ΔN residues along with their adjacent region, including a proline-rich P*XX*P motif [6]. High-throughput screens of p63 target genes revealed that p63 directly regulates nearly 7% of the coding genes in the genome, indicating complex interactions with many signaling pathways and differential effects on downstream biological responses. Although all p63 isoforms share the same DNA-binding domain, the composition of the functional p63 transcriptional complex seems to vary; thus, identifying specific interactors and modulators of p63 activity is of high importance in order to untangle the complexity of p63 function and design ΔNp63-targeted therapies for various diseases.

ΔNp63 exerts oncogenic properties and shows an oscillatory expression during cancer progression; ΔNp63 is generally overexpressed in differentiated primary epithelial tumors, whereas more aggressive and invasive tumors underexpress ΔNp63, which correlates with induction of an epithelial-to-mesenchymal transition (EMT) program, suggesting that ΔNp63 loss is crucial for tumor dissemination, acceleration of tumorigenesis and metastatic spread. However, once established, metastases at distant organs exhibit high ΔNp63 expression, indicating that ΔNp63 is required for extravasation and colonization [7–9]. In line with this, it has been recently demonstrated that ΔNp63 acts as a central transcriptional regulator of quasi-mesenchymal cancer stem cells (CSCs) that reside in an intermediate EMT state, driving colonization via autocrine epidermal growth factor (EGF) receptor (EGFR) signaling in breast cancer metastasis [10]. In both basal and luminal breast cancer models, ΔNp63 plays a prominent role in governing the tumor-initiating activity of cells by orchestrating the WNT, Hedgehog, BMP7 and NOTCH signaling pathways [10–15].

We have previously demonstrated that oncogenic RAS and transforming growth factor-β (TGFβ) signaling activate the ΔNp63 transcriptional program in breast and skin squamous cancer cells. In this context, ΔNp63 was shown to be critical for cell migration and invasion downstream of the EGF and TGFβ-SMAD signaling pathways through physical and functional interaction with the activator protein 1 (AP-1) family of transcription factors and the TGFβ receptor-regulated (R)-SMADs, i.e. SMAD2 and SMAD3 [16, 17].

Canonical TGFβ signaling is initiated by the phosphorylation-dependent activation of R-SMADs, by type I and type II kinase receptors (TGFβRI and TGFβRII, respectively), which enables the formation of complexes between R-SMADs and the common-partner (Co-) SMAD, SMAD4. The heteromeric R-SMAD-SMAD4 complexes translocate into the nucleus, where they regulate gene expression in cooperation with other transcription factors, co-activators and co-repressors [18, 19]. The TGFβ family of cytokines can regulate stem cell renewal and differentiation [20]. TGFβ-induced EMT correlates with the acquisition of stem cell-like properties and increased capability of sphere and tumor formation in vitro and in vivo, respectively [21, 22].

Several studies have linked p63 function to chromatin remodeling and enhancer reprogramming, especially during epidermal differentiation and stem cell maintenance [23, 24]. The p63 protein physically interacts with both BAF, an ATP-dependent nucleosome modifier and a member of the SWI/SNF complex, and KMT2D, a lysine-specific histone methyltransferase, controlling keratinocyte-specific open chromatin structure and expression of genes involved in epithelial development, adhesion, and differentiation [25, 26]. TGFβ signaling- and ΔNp63-mediated target gene regulation require interaction with different chromatin modifiers independent from each other [27]. In the current study, we show that TGFβ differentially affects the interaction between ΔNp63 and chromatin regulators, promoting chromatin accessibility and transcription of TGFβ/ΔNp63 target genes related to stemness and cell invasion.

## Methods

### Cell culture

MCF10A MII cells were obtained from Dr Peter ten Dijke (Leiden University, The Netherlands) and maintained at 37 °C and 5% CO_2_ in DMEM/F12 (Gibco, Life Technologies Ltd, Paisley, UK), supplemented with 5% fetal bovine serum (FBS) (Gibco, Life Technologies Ltd, Paisley, UK), 20 ng/ml EGF (PeproTech, EC Ltd, London, UK), 100 ng/ml cholera toxin (Sigma-Aldrich AB, Stockholm, Sweden), 0.5 µg/ml hydrocortisone (Sigma-Aldrich AB, Stockholm, Sweden), 10 µg/ml insulin (Sigma-Aldrich AB, Stockholm, Sweden and Gibco, Life Technologies Ltd, Paisley, UK). HCC1954 breast cancer cells, obtained from Dr Andrew J. G. Simpson (Ludwig Cancer Research, New York, USA), were maintained in RPMI-1640 (Sigma-Aldrich AB, Stockholm, Sweden and Gibco, Life Technologies Ltd, Paisley, UK), supplemented with 10% FBS and 100 U/ml penicillin and 100 mg/ml streptomycin (Sigma-Aldrich Sweden AB, Stockholm, Sweden) and 20 ng/ml EGF. The cell lines were frequently tested for the absence of mycoplasma and were authenticated by short tandem repeat analysis.

### TGFβ treatment and inhibitors

Recombinant human TGFβ1 (denoted TGFβ in this study) was purchased from PeproTech (EC Ltd, London, UK). Cells were starved overnight in a medium containing 0.2% serum (MCF10A MII) or 3% serum (HCC1954) before treatment with 5 ng/ml TGFβ. The following small molecule inhibitors were utilized at the indicated concentrations: TGFβRI kinase inhibitors (ALK5i) SB505124 (2.5 µM; Sigma-Aldrich AB, Stockholm, Sweden) and LY2157299 (2.0 µM; Sigma-Aldrich AB, Stockholm, Sweden), MEK1/2 inhibitor (MEKi) AZD6244 (0.25 µM; Selleckchem, Houston, TX 77230, USA), Jun N-terminal kinase inhibitor (JNKi) SP600125 (10 µM; Calbiochem, Merck, Stockholm, Sweden), p38 MAP-kinase inhibitor (p38i) SB203580 (10 µM; Tocris Bioscience, Bio-techne, Bristol, UK), and EZH2 inhibitor GSK343 (5 µM; Sigma-Aldrich AB, Stockholm, Sweden). All kinase inhibitors were dissolved in DMSO and added to the cells 20 min before TGFβ treatment. Protein synthesis was blocked by cycloheximide (CHX; C1988, Sigma-Aldrich AB, Stockholm, Sweden), administered to the cells at the same time as TGFβ treatment at a final concentration of 20 µg/ml.

### siRNA transfections

The ΔNp63 specific On-target plus SMART siRNA (sense sequence, 5´-GGACAGCAGCAUUGAUCAAUU; antisense sequence, 5´-UUGAUCAAUGCUGCUGUCCUU),

 the On-target plus Non-Targeting Control siRNA (Cat no: D-001810-01-20), the SUZ12 On-target plus siRNA pool (Cat no: L-006957-00-0005), the SMAD2 On-target plus siRNA pool (Cat no: L-003561-00-0005) and the SMAD3 On-target plus siRNA pool (Cat no: L-020067-00-0005) were purchased from Dharmacon (Horizon Discovery, Cambridge, UK). Stealth siRNAs specific for p63 (ID: HSS189462), SMAD2 (ID: VHS41107), SMAD3 (ID: VHS41111) and control siRNAs (Cat No. 12935-300 and 12935-200) were obtained from Invitrogen (Life Technologies, Ltd, Paisley, UK). siRNAs, at 20 nM (stealth) or 25 nM (siRNA pool) final concentration, were transfected using SiLentFect (Bio-Rad Laboratories AB, Solna, Sweden) transfection reagent according to the manufacturer’s instructions.

### Sphere formation assay

After siRNA transfection, HCC1954 cells (1 × 10^4^/well) were seeded in 96-well Costar ultra-low attachment plates (Corning, Corning, NY, USA) in RPMI medium supplemented with 20 ng/ml EGF and 10 ng/ml bFGF (Sigma-Aldrich AB, Stockholm, Sweden) and incubated with or without 5 ng/ml TGFβ for 8 days. Total sphere numbers per well (diameter > 50 μm) were counted under a microscope.

### Chromatin immunoprecipitation (ChIP)

ChIP was performed as previously described [16, 17]. In summary, cells were fixed in 1% formaldehyde, washed with ice-cold PBS, harvested by scraping, pelleted and resuspended in 1 ml of sodium dodecyl sulphate (SDS) lysis buffer (1% SDS, 50 mM Tris-HCl, pH 8.0, 10 mM EDTA, supplemented with Complete EDTA-free protease inhibitors (Roche Diagnostics, Scandinavia AB, Bromma, Sweden)). The cell lysates were subjected to sonication in a water bath using Diagenode Bioruptor sonicator (Diagenode, Bionordika, Stockholm, Sweden), with 30 s pulses for 5–10 min. Following sonication, samples were centrifuged at 14,000 rpm at 4 °C for 10 min. After removal of a control aliquot (whole-cell extract serving as an input), supernatants were diluted in ChIP dilution buffer (1% Triton X-100, 20 mM Tris-HCl, pH 8.0, 150 mM NaCl, 2 mM EDTA), and incubated at 4 °C overnight with antibodies precoupled to anti-mouse IgG or Protein A Dynabeads (Invitrogen, Life Technologies, Ltd, Paisley, UK) in PBS supplemented with 0.5% bovine serum albumin (BSA). The antibodies used for ChIP were raised against p63 (ab124762, Abcam, Cambridge, UK), H3K27ac (39685, Active motif, Carlsbad, CA and ab177178, Abcam, Cambridge, UK), H3K27me3 (61017, Active motif, Carlsbad, CA), p300 (61401, Active motif, Carlsbad, CA, and ab14984, Abcam, Cambridge, UK) and SUZ12 (39357, Active motif, Carlsbad, CA).

The precipitated complexes were washed five times in ChIP washing buffer (50 mM HEPES-KOH, pH 7.0, 0.5 M LiCl, 1 mM EDTA, 0.7% deoxycholate, 1% Igepal CA-630) and once with TE buffer (10 mM Tris-HCl, pH 8.0, 1 mM EDTA). Immunoprecipitated samples were eluted and reverse cross-linked at 65 °C in SDS lysis buffer. Genomic DNA was purified with a PCR purification kit (Qiagen, AB, Sollentuna, Sweden). The immunoprecipitated DNA was analyzed by qRT-PCR using locus-specific primers (the complete primer list can be found in Additional file 1-Table S1 in Supplementary Information) and normalized to the input DNA. The IgG control was included in all the experiments in order to check and confirm the specificity of the antibody used. The quantified relative fold change corresponded to the enrichment in each gene locus under treatment conditions divided by the enrichment in the control condition (control- or the sictrl-condition), as indicated.

### RNA isolation, cDNA synthesis and quantitative real-time-PCR

RNA was isolated by Total RNA Purification Kit (Norgen Biotek Corp, Canada). cDNA was prepared using High Capacity cDNA Reverse Transcription Kit (Applied Biosystems, Life Technologies, Ltd, Paisley, UK) utilizing 0.5 µg of total RNA, according to the manufacturer’s instructions. The cDNA samples were diluted 10 times in water. qRT-PCR was performed using 2× qPCR SyGreen Mix (PCR Biosystems, London, UK) and CFX96 real-time PCR detection system (Bio-Rad Laboratories AB, Solna, Sweden), according to the manufacturer’s instructions. Relative gene expression was determined using the ΔΔCt method. The expression was normalized to the *GAPDH* gene and quantified relative to the control condition. The complete primer list can be found in Additional file1-Table S2 in the Supplementary Information. Normalized mRNA expression levels are plotted in bar graphs that represent average values from triplicate determinations with standard deviations (SD). Each independent experiment was repeated at least three times.

### Nuclear/cytoplasmic fractionation

For mass spectrometry and co-immunoprecipitation analyses, nuclear and cytoplasmic fractions of MCF10A MII cells were separated after treatment or not with TGFβ (5 ng/ml) for 6 h. Briefly, cells were rinsed with PBS twice, scraped in PBS and centrifuged at 4^o^C for 5 min at 450×g. The cell pellet was resuspended in 50 mM Tris-HCl, pH 7.5, 10 mM MgCl_2_, 15 mM CaCl_2_, 1.5 M sucrose, complemented with 1% of protease inhibitor and 1% of 0.1 M of dithiothreitol (DTT). Cells were next incubated on ice for 15 min and 10% Igepal CA-630 was added before agitation and centrifugation for 30 s at 11,000×g. The supernatant contained the cytoplasmic fraction. The pellet was next resuspended in 50 µl of nuclear extraction buffer (20 mM HEPES, pH 7.9, 1.5 mM MgCl_2_, 0.42 M NaCl, 0.2 mM EDTA, 25% glycerol) complemented with 1% of protease inhibitor and 1% of 0.1 M DTT and agitated for 20 min at 4^o^C. Nuclear fraction was obtained as the supernatant after centrifugation for 5 min at 20,000×g at 4^o^C. Proteins were then quantified and subjected to SDS-polyacrylamide gel electrophoresis (SDS-PAGE).

### Co-immunoprecipitation (Co-IP) and immunoblotting analysis

For the co-immunoprecipitation assay, MCF10A MII cells treated or not with 5 ng/ml of TGFβ for 45 min or 6 h were lysed in lysis buffer (1% Triton X-100, 20 mM Tris-HCl, pH 7.5, 150 mM NaCl, 10% glycerol) and incubated overnight with anti-mouse IgG or protein A Dynabeads (Invitrogen, Life Technologies, Ltd, Paisley, UK) that had been preincubated with the indicated antibodies or mouse immunoglobulin G1 (IgG), (MAB002, R&D systems, Bio-techne, Minneapolis,MN, USA) or rabbit IgG (SouthernBiotech, Birmingham, AL, USA) in PBS, supplemented with 0.5% BSA. Following precipitation, the complexes were washed three times with lysis buffer and the immunoprecipitated proteins were eluted in 2× SDS Laemmli sample buffer, subjected to SDS-PAGE and blotted onto nitrocellulose membranes (Cytiva, Danaher, Uppsala, Sweden). The chemiluminescent signal was detected using the Immobilon Western kit (Merck Millipore, Stockholm, Sweden). For the immunoblotting analysis of total cell extracts, cells were lysed in 2× SDS Laemmli sample buffer (5% SDS, 25% glycerol, 150 mM Tris-HCl pH 6.8, 0.01% bromophenol blue, 100 mM DTT) prior to SDS-PAGE. The intensities of the bands from the chemiluminescent blot images of p-p63 (Ser66/68), p63 and tubulin from three independent experiments were quantified by Image lab 6.1 software and the intensity values of p63 bands were divided by the values of those of tubulin for the purposes of loading normalization. The normalized p63 values were then used to calculate the ratio of p-p63/p63 presented in the corresponding figures.

The antibodies used for co-IP and/or immunoblotting were raised against: phospho-Ser160/162 p63 (Ser 66/68 in ΔNp63) (#4981), SMAD3 (#9523), ERK1/2 (#4695), phospho-Thr202/Tyr204 ERK1/2 (#4370), phospho-c-JUN (Ser63) (#9261), p38 MAPK (#9212), phospho-p38 MAPK (Thr180/Tyr182) (#9211)  and Histone 3 (#9712) (Cell Signaling Technology, Danvers, MA, USA), SMAD2/3 (#610843) and cJUN (#610327) (BD Transduction Laboratories, Biosciences-Europe, Stockholm, Sweden), phospho-Ser465/467 SMAD2 (home-made [28]), Tubulin (T0198) (Sigma-Aldrich, AB, Stockholm, Sweden), DNMT1 (H-300, sc-20701) (Santa Cruz Biotechnology, California, USA), CHD4 (ab70469) and HDAC2 (ab51832) (Abcam Cambridge, UK) and NCOR2 (PAI-843) and HDAC3 (7G6C5) (Invitrogen, Life Technologies, Ltd, Paisley, UK).

### Mass spectrometry analysis

The nuclear fractions of MCF10A MII cells after treatment or not with TGFβ (5 ng/ml) for 6 h were subjected to immunoprecipitation with a p63 antibody immobilized on protein A Dynabeads. The complexes bound to beads were then subjected to mass spectrometry analysis at the Clinical Proteomics Mass Spectrometry Facility, Science for Life Laboratory, Karolinska Institutet, Sweden.

Briefly, on-bead reduction, alkylation and digestion (trypsin, sequencing grade modified, Pierce, Thermo Fischer Scientific, Sweden) was performed, followed by SP3 peptide clean-up of the resulting supernatant [29]. Each sample was separated using a Thermo Scientific Dionex nano LC-system in a 3 h 5–40% ACN gradient coupled to Thermo Scientific High Field QExactive. The software Proteome Discoverer vs. 1.4 including Sequest-Percolator for improved identification was used to search the *Homo sapiens* Uniprot database for protein identification, limited to a false discovery rate of 1%.

### Pathway enrichment analysis

The pathway enrichment analysis of the significantly enriched proteins was performed using the Enrich tool to query the gene ontology molecular function database. The UMAP dimensionality reduction method was applied to visualize the scatter plot of the enriched pathways utilizing the standalone enrichment analysis visualizer Appyter. The top significantly enriched molecular functions are indicated in Fig. 4 [30] .

The area under the curve (AUC) of the integrated signal intensity was used to quantify the relative abundance of each identified protein in the corresponding samples. The scaled AUC values were used for sample clustering and generation of heatmaps utilizing the pheatmap package in R.

### Statistical analysis

The figures and figure legends present the number of biological and technical replicates and the assessment of statistical significance. Data are presented as the mean ± SD from at least three independent experiments. Two-experimental group comparisons were performed using two-tailed unpaired Student’s *t*-test and multiple group comparisons were performed using the two-tailed unpaired Student’s *t*-test with Bonferroni correction. Statistical significance is represented by *p*-values *p ≤ 0.05, **p ≤ 0.01, ***p ≤ 0.001.

## Results

### TGFβ stimulation enhances p63 recruitment to DNA via TGFβRI/ALK5- and p38 MAP-kinase-dependent phosphorylation

**Fig. 1:**
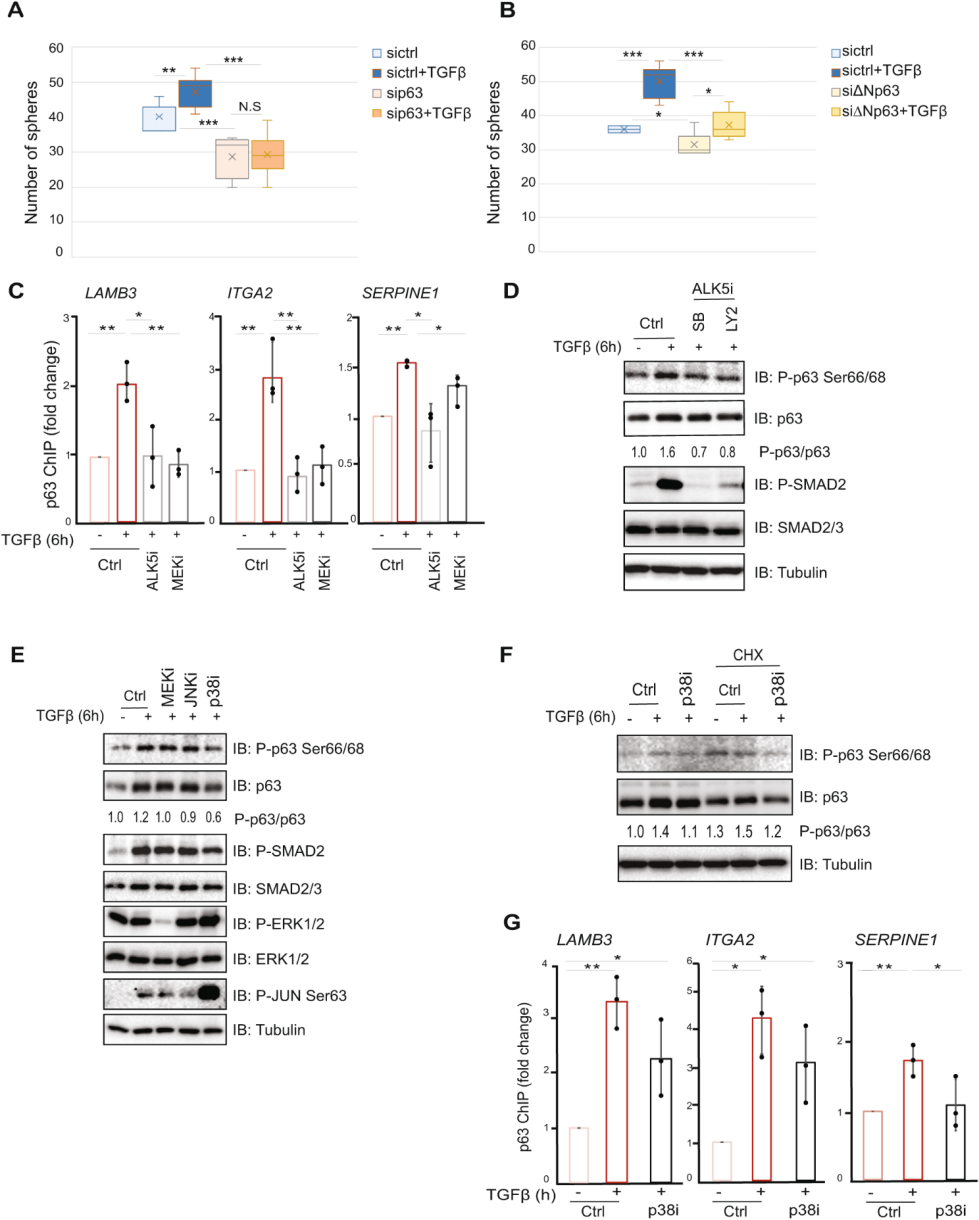
Activation of TGFβ signaling enhances p63 recruitment to DNA via ALK5/p38 kinase-dependent phosphorylation. **(A-B)** Sphere formation assay of HCC1954 cells in the presence or absence of TGFβ as indicated. Cells were transfected with non-targeting control (sictrl) siRNA or with siRNAs specific against all p63 isoforms **(A)** or specific against the ΔNp63 isoforms **(B)**. Cells were cultured in stem cell medium in 96-well ultra-low attachment plates. Sphere numbers per well were counted under microscopy. **(C)** ChIP-qPCR showing the effect of ALK5 kinase or MEK1/2 kinase inhibition on the TGFβ-induced recruitment of p63 to DNA. Values are expressed as relative fold-change corresponding to the enrichment of p63 antibody in each gene locus under treatment conditions divided by the enrichment in the control condition (ctrl). **(D-E)** TGFβ stimulation induced ALK5- and p38-dependent phosphorylation of ΔNp63 at Ser66/Ser68. Immunoblotting (IB) analysis of MCF10A MII cells, treated with the indicated kinase inhibitors or DMSO (ctrl) in the presence of TGFβ for 6 h. **(F)** Cycloheximide (CHX) chase experiment in MCF10A MII cells. Lysates of cells treated or not with p38 inhibitor (SB203580) or DMSO (ctrl) in the presence or not of TGFβ for 6 h were analyzed by IB with the indicated antibodies. In panels D-F, one of four independent experiments with similar results, is shown. The intensities of the p-p63 Ser66/68, p63 and tubulin bands from each of the four independent experiments were quantified and the values were used to calculate the ratio of p-p63/p63, presented between the corresponding immunoblots. **(G)** ChIP-qPCR showing the effect of p38 inhibition on the TGFβ-induced recruitment of p63 to DNA. Graphs presented in panels A-C and G show results of three independent experiments as mean ± SD; * *P* < 0.05, ** *P* < 0.01, *** *P* < 0.001. The dots in the graphs of panels C and G represent the individual values from each of the three independent ChIP experiments

The ΔNp63 and TGFβ transcriptional targets are involved in a network of signaling molecules that influence the stem cell niche [24]. In order to explore a possible role of ΔNp63 as an effector of TGFβ signaling in the regulation of stemness, we analyzed the sphere forming capacity of HCC1954 HER2^+^ breast cancer cells, which express only the ΔNp63α isoform, and virtually no TAp63 (EV, data not shown and [31, 32]), before and after ΔNp63 depletion, using siRNA against all p63 isoforms or ΔNp63 specific siRNA, and subsequent stimulation by TGFβ. We observed that, whereas TGFβ treatment increased the number of spheres, downregulation of ΔNp63 expression abrogated this effect, resulting in a significant reduction in the number of spheres (Fig. 1A, B).

We have previously demonstrated that the ΔNp63 activity downstream of TGFβ signaling in mammary epithelial cells is necessary for the regulated expression of several components of the extracellular matrix (ECM), such as laminin (LAMB3), integrin (ITGA2), plasminogen activator inhibitor-1 (PAI-1/SERPINE1), as well as heparin-binding EGF (HB-EGF) and EGFR that facilitate cell migration and invasion [16, 17]. We next sought to explore whether TGFβ treatment affects the recruitment of ΔNp63 to specific regions of the *LAMB3* (approximately 5 kbp upstream of the LAMB3 transcription start site (TSS)), *ITGA2* (approximately 2 kbp upstream of the *ITGA2* TSS) and *SERPINE1* (close to the *SERPINE1* TSS*)* gene loci. These regions have been previously identified as SMAD2/3- and ΔNp63-binding regions, based on SMAD2/3 and p63 ChIP seq analysis in the H-RAS-transformed MCF10A MII cells and HaCaT keratinocytes [16, 33, 34], both predominantly expressing the ΔNp63 isoform (Additional file 3, Fig. S1A) [35].

We found that TGFβ stimulation increased the binding of ΔNp63 to *LAMB3*, *ITGA2* and *SERPINE1* loci in MCF10A MII cells and in HCC1954 breast cancer cells without affecting ΔNp63 mRNA expression (Fig. 1C and Additional file 3, Fig. S1B, C). Furthermore, inhibition of TGFβ signaling by addition of a TGFβRI (also referred to as ALK5) kinase inhibitor or inhibition of the MEK1/2/ERK1/2 MAP-kinase (MAPK) pathway, by the AZD6244 inhibitor (MEKi), significantly decreased the binding of ΔNp63 to the *LAMB3*, *ITGA2* and *SERPINE1* gene loci (Fig. 1C). These data indicate that both TGFβ-SMAD and RAS-ERK1/2 MAPK signaling pathways regulate the DNA binding properties of ΔNp63, in agreement with previous findings [16, 17].

Next, we addressed the possible TGFβ-regulated mechanism enabling ΔNp63 activity. We found that treating MCF10A MII cells with TGFβ for 6 h resulted in an enhanced ΔNp63 phosphorylation at Ser66/Ser68 (Fig. 1D). In addition, we observed that TGFβ stimulation also induced phosphorylation of ΔNp63 at the same residues in breast cancer HCC1954 cells (Additional file 3, Fig. S1D). The increase in ΔNp63 phosphorylation was dependent on the kinase activity of TGFβRI/ALK5, since the addition of either of two different ALK5 kinase inhibitors (ALK5i, SB505124 and LY2157299) blocked this effect (Fig. 1D). We observed that the 1.6-fold induction by TGFβ stimulation in the ratio of p-p63/p63 was reduced to 0.7 and 0.8 after inhibition of the ALK5 kinase. This result is consistent with a previous study showing that TGFβ/ALK5 signaling can mediate ΔNp63 phosphorylation at the same sites [36].

TGFβ stimulation activates multiple downstream signaling pathways including the MEK1/2/ERK1/2, JNK and p38 MAPK pathways, which function cooperatively with the SMAD pathway in eliciting the TGFβ-induced physiological responses [37]. In order to elucidate the role of these pathways in the TGFβ-mediated phosphorylation of ΔNp63, we utilized specific inhibitors for each pathway. As shown in Fig. 1E and Additional file 3, Fig. S1E, F) the TGFβ-induced phosphorylation of ΔNp63 was quenched by inhibiting the kinase activity of p38 MAPK by SB203580 (p-p63/p63 ratio, 0.6), whereas the inhibition of either MEK1/2/ERK1/2 MAPK by AZD6244, or JNK MAPK by SP600125, showed no noticeable effect on ΔNp63 phosphorylation (p-p63/p63 ratio, 1 and 0.9 respectively). This result agrees with previous studies, where the p38 MAPK was found to mediate p63 phosphorylation [38–40]. The p38 activation at 6 h after TGFβ stimulation in MCF10A MII cells was not dependent on the ALK5 kinase activity or SMAD2/3 activation consistent with previous observations (Additional file 3, Fig. S1F, G) [41, 42]. We next investigated the effect of p38-induced ΔNp63 phosphorylation on the stability and DNA binding activity of ΔNp63. We observed that TGFβ stimulation slightly increased the stability of p63, as analyzed by CHX treatment (p-p63/p63 ratio from 1.3 to 1.5), whereas inhibition of the p38 kinase led to destabilization of ΔNp63 protein (p-p63/p63 ratio, 1.2) (Fig. 1F) and reduced binding of ΔNp63 to the *SERPINE1* gene locus (Fig. 1G).

Altogether, these results indicate that activation of the TGFβ signaling pathway promotes the DNA binding properties of ΔNp63 through ALK5- and p38-induced phosphorylation at Ser66/Ser68 and subsequent stabilization of ΔNp63.

### ΔNp63 orchestrates remodeling of histone marks in response to TGFβ stimulation

In order to explore the possible involvement of the TGFβ pathway in chromatin organization and the interplay between histone modification marks, we performed ChIP analysis using MCF10A MII cells and antibodies against K27 acetylation (K27ac) and K27 tri-methylation (K27me3) of Histone 3 (H3); these modifications are mutually exclusive and are associated with active or inactive gene transcription, respectively.

**Fig. 2:**
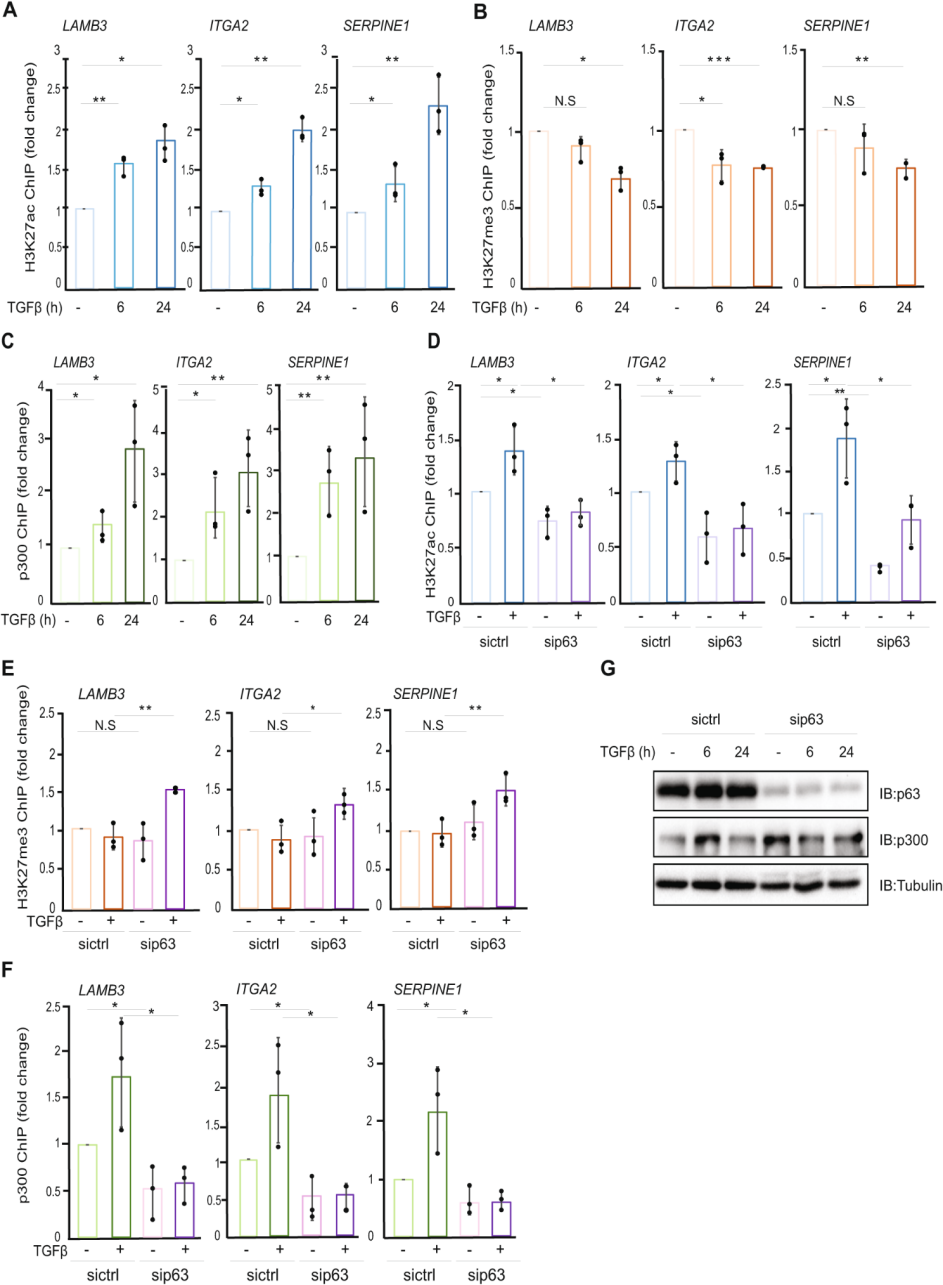
TGFβ-induced gene expression correlates with changes in histone modification marks orchestrated by p63. **(A-B)** ChIP-qPCR showing the changes in H3K27ac **(A)** and H3K27me3 **(B)** histone marks of the indicated gene loci in MCF10A MII cells, incubated in starvation medium overnight and stimulated or not with TGFβ for the indicated time-periods. **(C)** ChIP-qPCR showing the effect of TGFβ treatment on p300 binding to the indicated gene loci in MCF10A MII cell. **(D-E)** ChIP-qPCR experiments showing the effect of p63 depletion on H3K27ac **(D)** and H3K27me3 **(E)** marks. MCF10A MII cells transfected with non-targeting control (sictrl) siRNA or with siRNA specific against all p63 isoforms were incubated overnight in starvation medium and treated or not with TGFβ for 24 h. **(F)** Effect of p63 depletion on p300 recruitment to chromatin. MCF10A MII cells transfected with siRNAs and treated or not with TGFβ as in panels D and E, were subjected to ChIP with p300 antibody and subsequent qPCR analysis. **(G)** Effect of p63 depletion on p300 expression. MCF10A MII cells transfected with non-targeting control (sictrl) siRNA or with siRNAs specific against all isoforms of p63 were treated or not with TGFβ for 6–24 h and subjected to IB analysis with the indicated antibodies. Data are representative of three independent experiments with similar results. Graphs presented in panels A-F show results of three independent experiments as mean ± SD; * *P* < 0.05, ** *P* < 0.01, *** *P* < 0.001, N.S: not significant difference. The dots represent the individual values from each of the three independent experiments

We found that, consistent with changes in transcription of the extracellular matrix genes (Additional file 3, Fig. S2A), TGFβ treatment increased the levels of H3K27ac at the specific SMAD2/3- and p63-binding regions of the *LAMB3*, *ITGA2* and *SERPINE1* genes (Fig. 2A). At the same genomic regions, TGFβ stimulation slightly decreased the repressive H3K27me3 mark (Fig. 2B). Additionally, the expression of *LAMB3*, *ITGA2* and *SERPINE1*, as well as the levels of the active transcription mark H3K27ac, were dramatically decreased by treating the cells with the MEK1/2 inhibitor AZD6244 (Additional file 3, Fig. S2A, B), confirming the role of EGF-RAS-MEK1/2-ERK1/2 signaling in transcriptional regulation of TGFβ/ΔNp63 target genes. The decreased H3K27ac levels at the investigated loci upon MEK1/2/ERK1/2 inhibition were accompanied by increased deposition of the H3K27me3 mark and enhanced recruitment of Suppressor of Zeste-12 protein (SUZ12), a component of the Polycomb Repressive Complex 2 (PCR2) complex [43], which catalyzes the tri-methylation on H3K27 (Additional file 3, Fig. S2C, D). These results suggest that the MEK1/2-ERK1/2 pathway is important for depositing active chromatin marks and these changes in the histone landscape mediate the TGFβ- and MEK/ERK1/2-dependent transcriptional effects.

We next examined the effect of TGFβ stimulation on the recruitment of p300 on the TGFβ/SMAD/ΔNp63 gene loci. The acetyltransferase p300 catalyzes the acetylation of H3K27 and has been previously found to interact physically with SMAD3 and SMAD4 [44], promoting SMAD3 transcriptional activity by catalyzing its acetylation [45]. ChIP-qPCR analysis using MCF10A MII cells showed that TGFβ treatment significantly increased the binding of p300 to the *LAMB3*, *ITGA2* and *SERPINE1* regions (Fig. 2C).

As a master regulator of cell differentiation, p63 regulates transcriptional programs via chromatin remodeling at its target genes [46, 47]. Therefore, we next examined whether ΔNp63 is sufficient to promote H3K27ac on its transcriptional targets. We observed that silencing of ΔNp63 decreased both the basal and the TGFβ-induced H3K27 acetylation on *LAMB3*, *ITGA2* and *SERPINE1*, whereas H3K27 tri-methylation increased in the same regions (Fig. 2D, E, Additional file 3, Figs. S2E, F, H). In agreement with these changes, depleting MCF10A MII cells of ΔNp63 resulted in a significant reduction of p300 binding to DNA (Fig. 2F and Additional file 3, Fig. S2G). Moreover, we confirmed that p63 silencing had no significant effect on p300 expression levels (Fig. 2G and Additional file 3, Fig. S2I).

### TGFβ stimulation recruits a ΔNp63-p300 complex to target gene loci

**Fig. 3:**
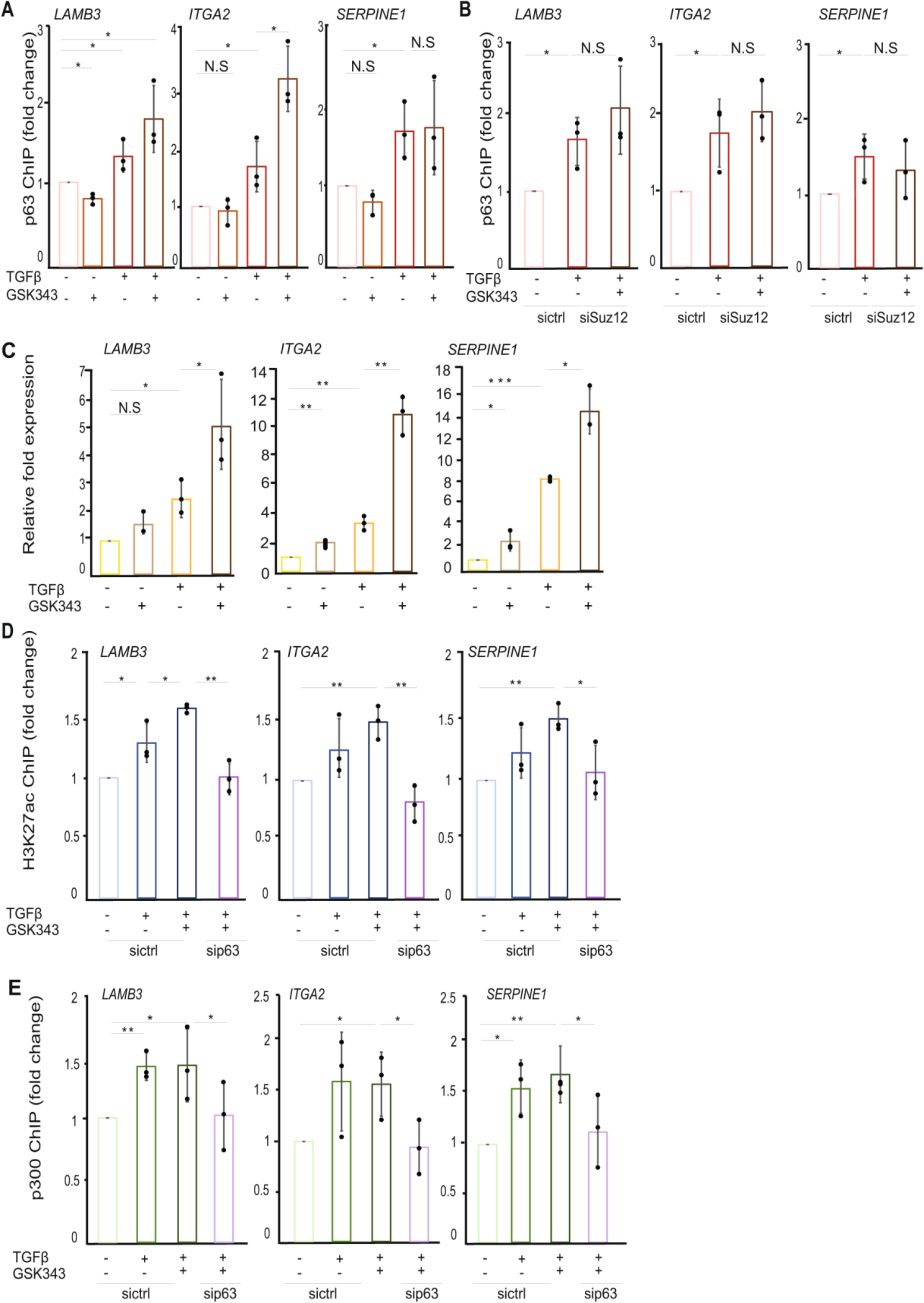
H3K27me3 inhibition increases p63 and p300 recruitment to chromatin only in the presence of active TGFβ signaling. **(A)** ChIP-qPCR showing the recruitment of p63 to the indicated gene loci in MCF10A MII cells treated or not with an EZH2 inhibitor (GSK343) for 48 h in the presence or not of TGFβ stimulation for 24 h. **(B)** ChIP-qPCR showing the effect of SUZ12 depletion in combination with treatment with a GSK343 inhibitor on the TGFβ-induced p63 binding to the indicated gene loci. **(C)** qRT-PCR analysis of the effect of TGFβ stimulation and GSK343 inhibition on the expression of *LAMB3*, *ITGA2* and *SERPINE1* genes. MCF10A MII cells were incubated overnight in starvation medium and subsequently treated with 5 µΜ GSK343 inhibitor for 24 h before the stimulation or not with TGFβ for an additional 24 h. **(D, E)** Effect of p63 silencing on the H3K27ac mark and the recruitment of p300 in MCF10A MII cells treated or untreated with TGFβ and GSK343. Lysates of MCF10A MII cells were subjected to ChIP with H3K27ac **(D)** of p300 **(E)** antibodies and subsequent qPCR analysis. Graphs presented in panels A-E show results of three independent experiments as mean ± SD; * *P* < 0.05, ** *P* < 0.01, ***P < 0.001, N.S: not significant difference. The dots represent the individual values from each of the three independent experiments

To further investigate the sequence of the described events, we used GSK343, a selective EZH2 inhibitor, in order to block the tri-methylation of H3K27 and determine its functional relevance for ΔNp63/p300 and subsequent histone modifications. As shown in Additional file 3, Fig. S3A, B, treatment of MCF10A MII cells with GSK343 for 48 h efficiently decreased H3K27me3, whereas it increased H3K27ac, on *LAMB3 and ITGA2* loci. Inhibition of H3K27me3 increased the TGFβ-induced recruitment of ΔNp63 only to the *ITGA2* locus, without significant effect on the *LAMB3* and *SERPINE1* loci (Fig. 3A). These data support the notion that changes in the chromatin accessibility are not sufficient to recruit ΔNp63, and that TGFβ stimulation is an essential upstream directing signal. The EZH2 inhibition results were further confirmed by depleting a second PRC2 subunit, SUZ12. Upon SUZ12 depletion or GSK343 treatment, decreased levels of H3K27me3 were accompanied with increased H3K27ac levels (Additional file 3, Fig. S3C); these changes had only slight effect on the TGFβ-induced recruitment of ΔNp63 to its target genomic regions (Fig. 3B). Consequently, upregulation of expression of *LAMB3*, *ITGA2* and *SERPINE1* was significantly higher after combined treatment with TGFβ and GSK343, compared to individual treatments (Fig. 3C).

In summary, using of two different approaches, inhibition of the EZH2 methyltransferase activity of the PRC2 complex and depletion of SUZ12, we confirmed that the activation of the TGFβ pathway acts as an upstream signal directing ΔNp63 to its target gene regions enabling their transcriptional upregulation.

We next investigated whether ΔNp63 is necessary to establish the H3K27ac mark on the TGFβ/ΔNp63 gene loci. Interestingly, we noticed that the activation of the TGFβ pathway, even combined with EZH2 inhibition, did not enhance the H3K27ac levels or the recruitment of p300 to the indicated gene loci upon ΔNp63 depletion (Fig. 3D, E). These results emphasize the indispensable role of ΔNp63 in recruiting histone modifying enzymes, such as p300, to their target genomic regions, regulating chromatin accessibility and gene transcription.

### ΔNp63 interacts with several components of the epigenetic machinery

**Fig. 4:**
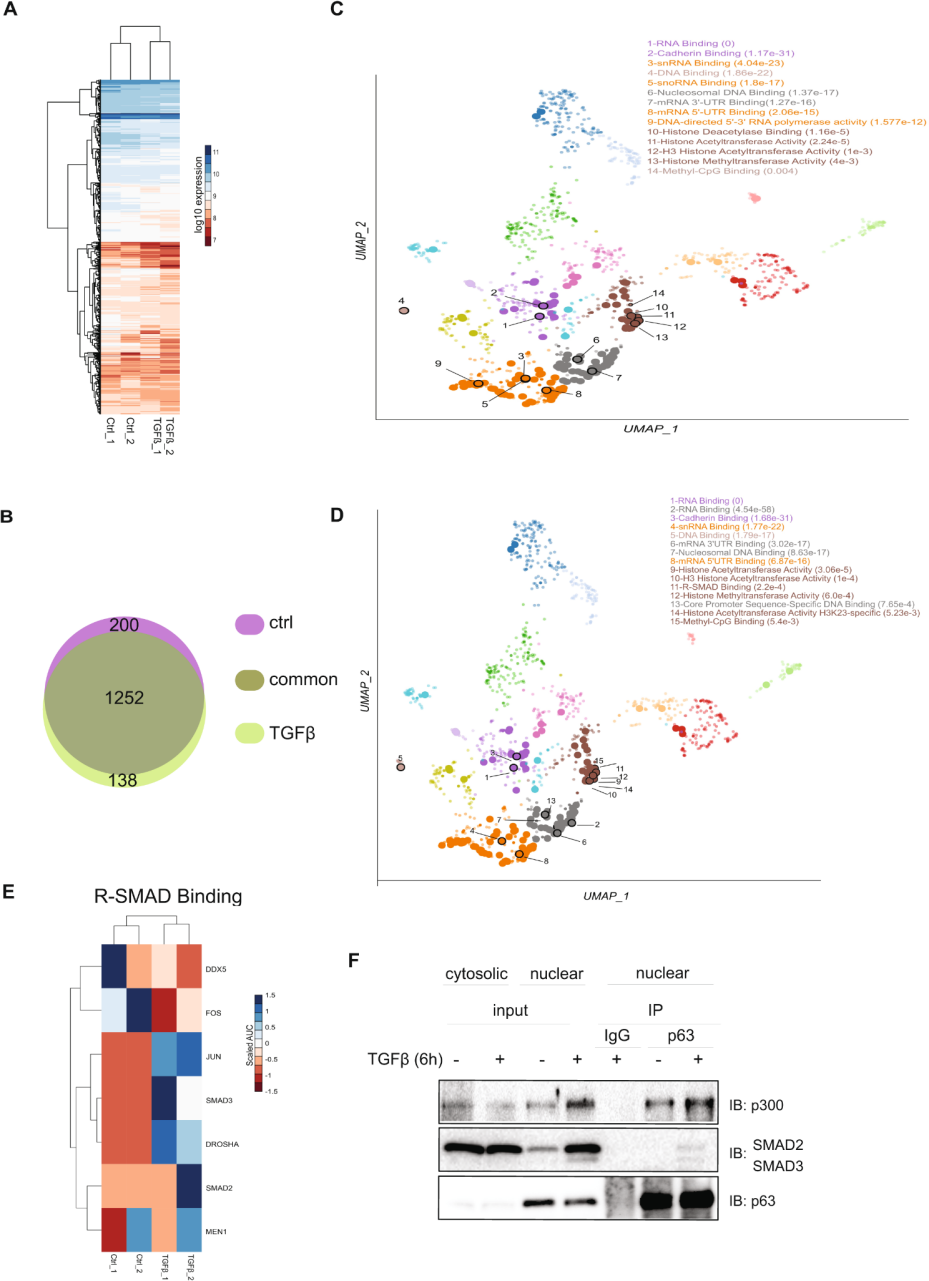
Identification of ΔNp63-interacting proteins. **(A)** Heatmap showing the peptide intensities derived from the MS/MS spectrum for control samples (Ctrl) and TGFβ-treated samples. Log10 expression represents peptide intensities derived from quantifying area under curve (AUC) of significantly enriched peaks. **(B)** Illustration of the number of the identified p63 interactors, enriched in both control condition and TGFβ-treated condition (common), uniquely enriched in control condition (control) or uniquely enriched in TGFβ-treated condition (TGFβ). **C, D** UMAP plots visualizing the most significantly enriched molecular functions derived from the gene ontology database and associated with proteins identified in control samples **(C)** and TGFβ-treated samples **(D)**. **(E)** Heatmap depicting the intensity patterns of proteins involved in R-SMAD binding in the respective samples. Scaled AUC was calculated using Z-score method representing quantified AUC of peptide intensities. **(F)** p63 interaction with p300 and SMAD2/3. MCF10A MII cells, starved in 0.2% FBS medium and stimulated with TGFβ or not for 6 h, were subjected to nuclear-cytosolic fractionation. The nuclear lysates (input nuclear) were immunoprecipitated (IP) with p63-specific antibody, or IgG control, and analyzed by immunoblotting utilizing specific antibodies, as indicated. One of three independent experiments with similar results, is shown

The interaction between ΔNp63 and different proteins specifies the transcriptional regulation of various target genes [48]. To identify new ΔNp63 interactors and to explore the effect of activation of the TGFβ pathway on the ΔNp63 interactome, we performed mass spectrometry analysis using MCF10A MII cells. Specifically, nuclear extracts isolated from cells treated or not with TGFβ for 6 h were subjected to immunoprecipitation with a p63 antibody and subsequent proteomic analysis (Additional file 3, Fig. S4A). Based on the profile of all detected proteins, we observed a proper clustering of the control versus TGFβ-treated samples (Fig. 4A). Among the identified proteins interacting with ΔNp63, the majority of hits (1252 proteins) was detected in both untreated (ctrl) and TGFβ-treated conditions (Fig. 4B, Suppl. Figure 4B). However, certain ΔNp63-interacting proteins were uniquely enriched either in the untreated condition (ctrl) (200 proteins) or after activation of the TGFβ pathway (138 proteins) (Fig. 4B and Suppl. Figure 4B). We next analyzed and categorized the ΔNp63 interactors based on their molecular function; the top enriched processes for control and TGFβ-stimulated conditions are represented in the UMAP plots (Fig. 4C and D, respectively). Notably, we observed interaction between ΔNp63 and SMAD3 transcription factors only in the TGFβ-treated condition, confirming the fidelity of our sample preparation and TGFβ treatment (Fig. 4D, E). Furthermore, the detection of proteins that have been previously described to interact with p63, such as p300 and the AP-1 family transcription factor JUNB, strengthened the validity of our mass spectrometry results [17, 49, 50] (Fig. 4E, Additional file 3, Fig. S4C). Interestingly, both before and after TGFβ stimulation, we observed high enrichment in processes related to histone remodeling and chromatin binding (Fig. 4C, D; the next section describes specific protein hits), consistent with recent studies that have implicated ΔNp63 in reprogramming of enhancers and shaping the chromatin landscape in different tumor types [23, 46, 47]. Also, the data agree with earlier studies suggesting that ΔNp63 functions as a pioneer transcription factor that targets its binding sites within inaccessible chromatin and induces chromatin remodeling [51]. We next performed co-immunoprecipitation experiments in order to confirm the interaction of ΔNp63 with the SMAD2 and SMAD3 transcription factors and the acetyltransferase p300. Interestingly, we observed that activation of TGFβ signaling induced the interaction of ΔNp63 with p300 and SMAD2/3 (Fig. 4F). Taken together, our data demonstrate the role of TGFβ signaling in promoting novel protein interactions of ΔNp63.

### Activation of TGFβ signaling switches the ΔNp63 epigenetic interactors

**Fig. 5:**
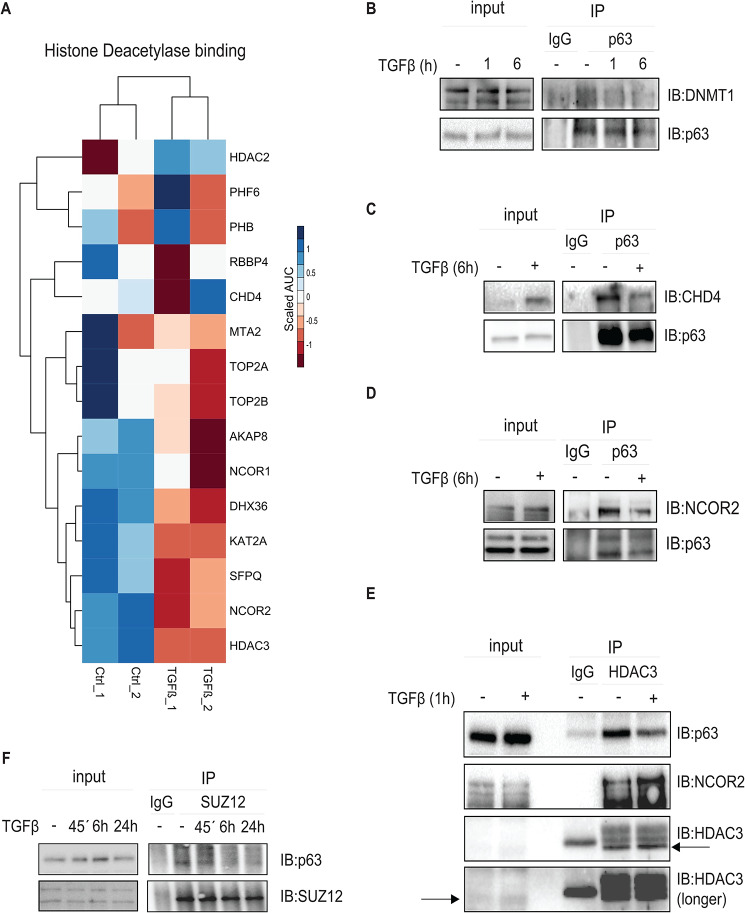
Activation of TGFβ signaling induces a switch in the ΔNp63 epigenetic interactors. **(A)** Heatmap depicting the intensity patterns of proteins involved in histone deacetylase binding in the respective samples. Scaled AUC was calculated using Z-score method representing quantified AUC of peptide intensities. **(B-E)** MCF10A MII cells, starved in 0.2% FBS medium and stimulated with TGFβ or not for the indicated time points, were subjected to immunoprecipitation (IP) with a p63-specific antibody **(B-D)** or an HDAC3-specific antibody **(E)**, or IgG control, and analyzed by IB with the antibodies recognizing p63 **(B-E)** and DNMT1 **(B)**, CHD4 **(C)**, NCOR2 **(D)** or HDAC3 **(E)**. In E, arrows indicate the band detecting HDAC3 expression. **(F)** SUZ12 interaction with p63. MCF10A MII cells, starved in 0.2% FBS medium and stimulated with TGFβ for the indicated time periods, were subjected to IP with SUZ12-specific antibody and subsequent IB with specific antibodies. In panels B-F, one of three independent experiments with similar results, is shown

Among the identified ΔNp63 epigenetic interactors, the group of proteins that are functionally involved in histone deacetylase binding and histone acetyltransferase activity showed higher statistical significance in the untreated (ctrl) condition compared to stimulated samples (Fig. 5A). Interestingly, we detected ΔNp63 interactions with several members of the Nucleosome Remodeling and Deacetylase (NURD) complex, including the ATP-helicase CHD4, the histone deacetylase HDAC2, the histone chaperone RBBP4 and the metastasis-associated protein MTA2. Additional interactions were detected with the NCOR/SMRT HDAC3 complex, including the histone deacetylase HDAC3 and the nuclear receptor corepressors 1 and 2 (NCOR1 and NCOR2) (Fig. 5A). The subunits of these two histone-modifying complexes, well known to be involved in histone deacetylation and inactivation of gene transcription, were identified in the present study as novel ΔNp63 chromatin interactors. Additionally, our mass spectrometry analysis followed by co-immunoprecipitation experiments validated a novel interaction of ΔNp63 with the DNA methyltransferase 1 (DNMT1) which was decreased upon TGFβ stimulation (Fig. 5B and Additional file 3, Fig. S4C). DNMT1 has previously been described to interact with the CHD4 component of the NURD complex during DNA damage induced by oxidative stress, and to help the maintenance of DNA hypermethylation-associated transcriptional silencing of tumor suppressor genes [52].

We performed co-immunoprecipitation analysis to validate the interaction of ΔNp63 with the NURD components CHD4 and HDAC2 (Fig. 5C and Additional file 3, Fig. S5A), as well as with the NCOR/SMRT HDAC3 members, NCOR2 and HDAC3 (Fig. 5D, E and Additional file 3, Fig. S5B). Interestingly, while the activation of TGFβ signaling strongly induced the interaction of ΔNp63 with p300 and SMAD2/3 (Fig. 4F), TGFβ significantly reduced the interaction between ΔNp63 and CHD4, NCOR2, HDAC3 and DNMT1 (Fig. 5B-E). However, the ΔNp63 interaction with HDAC2 was not significantly affected upon TGFβ stimulation (Additional file 3, Fig. S5A). We also investigated the possible interaction between ΔNp63 and the PRC2 complex, specifically with SUZ12 which has been previously found to interact with CHD4 and to be recruited by CHD4 to specific genomic regions [53, 54]. We detected an interaction between SUZ12 and ΔNp63 (Fig. 5F). Moreover, TGFβ treatment for 6 h led to the dissociation of the ΔNp63-SUZ12 complex.

In summary, these results confirm the data obtained from the mass spectrometry analysis and suggest that TGFβ differentially affects the interaction of ΔNp63 with the chromatin remodeling complexes. Activation of TGFβ signaling decreased interaction between ΔNp63 and the histone deacetylase complexes CHD4/NURD, NCOR/SMRT/ HDAC3 and SUZ12, whereas it increased the formation of complexes between ΔNp63, SMAD2/3 and p300.

### Activation of SMAD2 and SMAD3 transcription factors drives the ΔNp63 selectivity on histone-modifying complexes

**Fig. 6:**
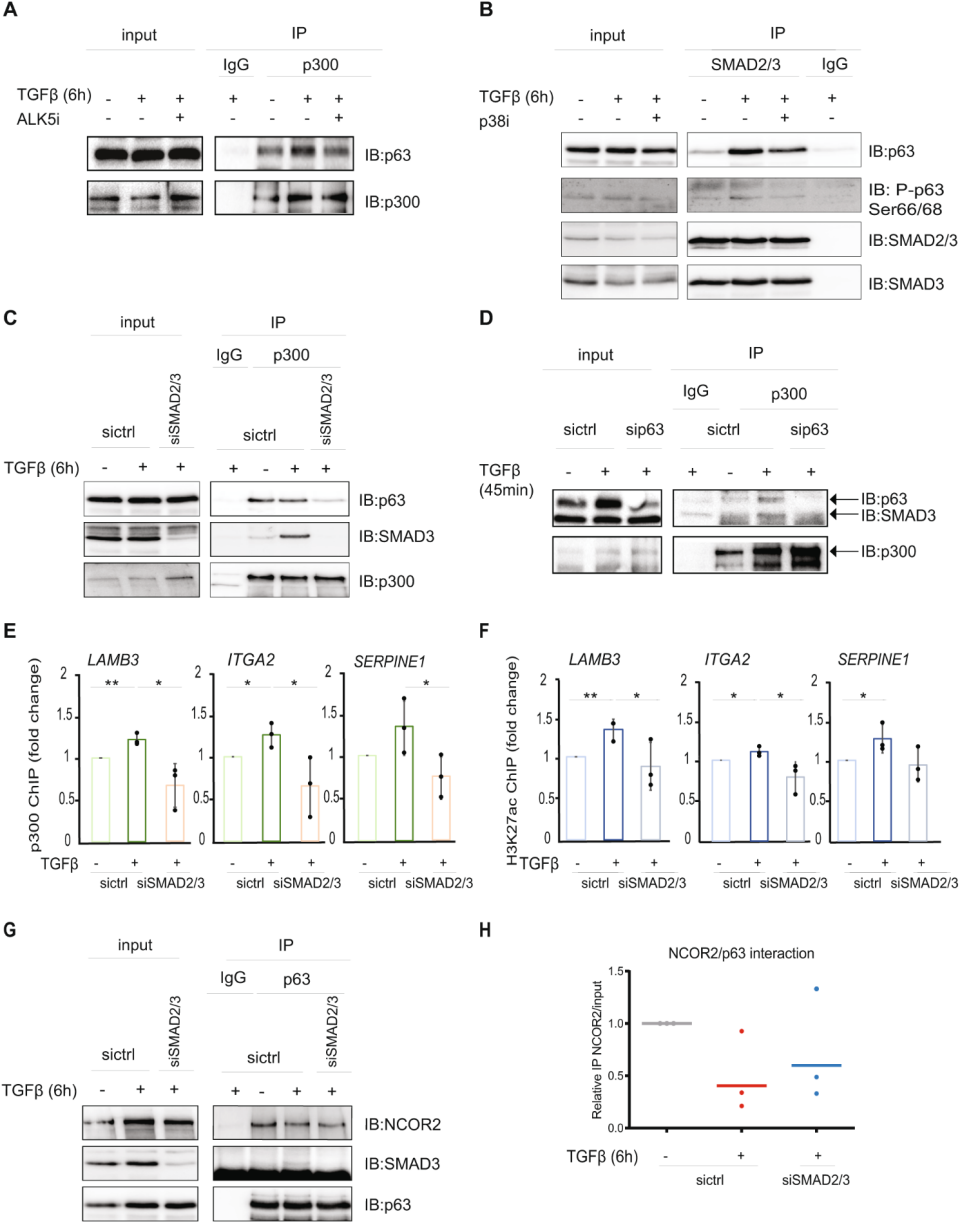
Activation of SMAD2 and SMAD3 transcription factors drives the p63 selectivity on histone modulation complexes. **(A)** Effect of ALK5 inhibition on p63/p300 interaction. Lysates of MCF10A MII cells treated with ALK5 kinase inhibitor (SB505124) or not (control) in the presence of TGFβ stimulation were subjected to IP with p300-specific antibody or IgG control, and analyzed by IB with the indicated antibodies. (**B**) Effect of p38 inhibition on p63/SMAD2/3 interaction. Lysates of MCF10A MII cells treated with p38 kinase inhibitor (SB203580) or not (control) in the presence of TGFβ stimulation were subjected to IP with SMAD2/3 antibody or IgG control, and analyzed by IB with the indicated antibodies. **(C)** Effect of SMAD2/3 depletion on p63/p300 interaction. MCF10A MII cells transfected with non-targeting control (sictrl) siRNA or with siRNA specific against SMAD2 and SMAD3 were incubated in starvation medium and treated or not with TGFβ for 6 h. Cell lysates were subjected to IP with p300-specific antibody or IgG control, and analyzed by IB with the indicated antibodies. **(D)** Effect of p63 depletion on SMAD3/p300 interaction. MCF10A MII cells transfected with non-targeting control (sictrl) siRNA or with siRNA specific against all p63 isoforms were incubated in starvation medium overnight and treated or not with TGFβ for 45 min. Cell lysates were subjected to IP with a p300-specific antibody or IgG control, and analyzed by IB with the indicated antibodies. **(E**,** F)** Effect of SMAD2/3 depletion on p300 recruitment to chromatin **(E)** and H3K27ac **(F)**. MCF10A MII cells transfected with siRNAs and starved as in panel C were treated or not with TGFβ for 24 h. Cell lysates were subjected to ChIP with p300 **(E)** or H3K27ac **(F)** antibodies and subsequent qPCR analysis. Graphs presented in panels E-F show results of three independent experiments as mean ± SD; * *P* < 0.05, ** *P* < 0.01. The dots represent the individual values from each of the three independent experiments. **(G)** Effect of SMAD2/3 depletion on p63/NCOR2 interaction. MCF10A MII cells transfected with siRNAs and starved as in panel A were treated or not with TGFβ for 6 h. Cell lysates were subjected to IP with p63 and analyzed by IB with the indicated antibodies. One of three independent experiments with similar results, is shown. (**H**) Graph illustrating the relative IP NCOR2/input as average values from the quantification of three independent experiments performed as described in panel G. The dots represent the individual values from each of the three independent experiments

We demonstrated that TGFβ stimulation enhanced the DNA binding properties of ΔNp63 and caused the dissociation of the ΔNp63/NURD and ΔNp63/NCOR/SMRT HDAC3 complexes, while promoting the interaction of ΔNp63 with the acetyltransferase p300. In order to examine the mechanism by which TGFβ enables the complex formation between ΔNp63 and p300, we first utilized the TGFβRI/ALK5 kinase inhibitor and performed co-immunoprecipitation experiments in MCF10A MII cells. Inhibition of the ALK5 kinase activity counteracted the increase in the ΔNp63-p300 interaction promoted by TGFβ treatment (Fig. 6A). We also investigated whether the p38-dependent phosphorylation of ΔNp63 affected the association with its interactors, SMAD2/3 and p300. As shown in Fig. 6B and Additional file 3, Fig. S6A, inhibition of p38 activity resulted in reduced interaction of ΔNp63 with SMAD2/3 and p300, as well as of p300 with SMAD2/3; these observations confirm the importance of TGFβ-induced phosphorylation and subsequent stabilization of ΔNp63 in facilitating ΔNp63/SMAD2/3/p300 protein complex formation and activation of transcription. Our findings are consistent with the notion that p38 phosphorylates ΔNp63 and that the ALK5-dependent phosphorylation of SMAD2/3 promotes their nuclear localization, facilitating interaction with phosphorylated ΔNp63. Detailed mechanisms by which the TGFβ/p38-dependent phosphorylation of ΔNp63 [36, 40] is regulated in the nucleus require further investigation.

As demonstrated in previous studies, the phosphorylation-induced conformational change of SMAD3 regulates its association kinetics with p300 [55]. We, therefore, investigated whether the presence of activated SMAD2 and SMAD3 facilitates the interaction between ΔNp63 and p300. We noticed that the depletion of both SMAD2 and SMAD3 dramatically reduced the TGFβ-induced ΔNp63/p300 interaction (Fig. 6C and Additional file 3, Fig. S6B). We also explored whether ΔNp63 is needed for the interaction between SMAD2/3 and p300. As shown in Fig. 6D and Additional file 3, Fig. S6C, ΔNp63 knockdown appreciably decreased the association between SMAD2/3 and p300. In line with these findings, ChIP-qPCR analysis showed that the recruitment of p300 to the *LAMB3*, *ITGA2* and *SERPINE1* gene loci was inhibited upon SMAD2 and SMAD3 knockdown (Fig. 6E). As a consequence of the reduced p300 recruitment to the TGFβ/ΔNp63 target gene loci, the levels of H3K27ac mark were lower upon SMAD2 and SMAD3 depletion (Fig. 6F). In summary, our data suggest that activation of TGFβ signaling leads to complex formation between ΔNp63, SMAD2/3 and p300, facilitating chromatin remodeling and gene transcription.

Since TGFβ stimulation promoted the formation of a complex between SMAD2/3, ΔNp63 and p300, and the phosphorylation of SMAD2/3 was required for the interaction between ΔNp63 and p300, we further explored whether the presence of SMAD2/3 affects the selectivity between the ΔNp63 interactors. By comparing the interaction of ΔNp63 with NCOR2 in the presence or absence of SMAD2/3, we observed that while SMAD2/3 depletion decreased the ΔNp63-p300 association (Fig. 6C), it also enhanced the ΔNp63-NCOR2 interaction (Fig. 6G, H). This result was confirmed after quantification of the three independent experiments (Fig. 6H), since we observed that in the current context, TGFβ stimulation caused reproducible upregulation of NCOR2 expression resulting in input fluctuations between the conditions.

Together, these results indicate that activation of the TGFβ effectors SMAD2 and SMAD3 controls the interaction of ΔNp63 with different histone modulators, pointing towards the dissociation of ΔNp63 and histone deacetylation complexes, and formation of a ΔNp63-p300 complex. This dynamic shift in the balance between histone deacetylation and acetylation enables the opening of the chromatin and the transcriptional regulation of the TGFβ/ΔNp63 target genes.

## Discussion

Epigenetic regulation enables cells to sense and respond to growth factor signaling in a cell context-dependent manner. The interplay between TGFβ signaling and the epigenetic machinery serves as a versatile fine-tuning mechanism regulating gene transcription during biological processes involved in embryonic development and disease progression, particularly cancer. We have previously shown that after short periods of TGFβ stimulation, SMAD2/3 factors preferentially bind to enhancer regions already accessible in normal mammary epithelial cells, whereas after longer treatment with TGFβ, the SMAD2/3 complex relocates to different genomic regions [33]. This observation indicates that the recruitment of SMAD2/3 to genomic sites associates with regulated chromatin accessibility and architecture. Indeed, the current study shows that TGFβ-induced transcription of target genes positively correlates with changes in histone modification marks, accompanied by enhanced recruitment of the acetyltransferase p300 and the transcription factor ΔNp63. In line with our data, it has been recently demonstrated that, in epithelial cells, TGFβ promotes widespread enhancer chromatin opening and that the TGFβ-activated enhancers are strongly enriched in SMAD2/3/4 and AP-1 footprints [27]. Activated SMAD complexes can recruit various epigenetic regulators, such as histone modifiers, DNA modifiers, nucleosome remodelers, and lncRNAs, to regulate the transcription of cell context-dependent TGFβ signaling target genes. The presence of SMAD transcription factors in chromatin-remodeling complexes provides an opportunity to targeted treatment of tumors with active TGFβ signaling [56].

SMAD complexes have a weak affinity to DNA; thus, being driven to cooperate with other site-specific transcription factors or pioneer factors that actively recruit the SMAD complexes or stabilize their DNA binding [57]. Pioneer transcription factors can bind directly to condensed chromatin and are essential in recruiting other transcription factors and histone modifying enzymes, as well as controlling DNA methylation. Our previous work showed that ΔNp63, among other transcription factors, binds SMAD2/3 and AP-1 family proteins and regulates their recruitment to the TGFβ target gene loci [17]. Moreover, we and others have also found that ΔNp63 interacts with the acetyltransferase p300 and the SNF-SWF-BAF chromatin remodeling complex [25], while co-occupancy by p63 was observed in approximately 50% of the DNA methyltransferase 3a (DNMT3a)-bound enhancers in epidermal stem cells. These target enhancers, where p63 depletion reduces DNMT3a localization, associate with the expression of genes involved in keratinocyte proliferation and cellular identity specification [58]. Based on these findings, p63 exerts intrinsic pioneer factor activity and together with co-regulating transcription and chromatin factors, such as BAF [4, 25, 59], bookmarks dynamic enhancers and regulates chromatin accessibility. We indeed highlighted that ΔNp63 expression is sufficient to induce a switch in the ratio of the mutually exclusive histone modification marks, H3K27ac and H3K27me3, on specific TGFβ/SMAD regulated gene loci by recruiting the acetyltransferase p300.

On the other hand, since the inhibition of H3K27me3 had no significant effect on ΔNp63 binding to DNA, it is likely that TGFβ is the upstream signal responsible for recruiting ΔNp63 to the target genomic loci. We demonstrated that TGFβ stimulation, through p38 MAPK activation, induces phosphorylation of ΔNp63 at Ser66/68 leading to enhanced ΔNp63 protein stability and DNA binding properties. Given that *TP63* mutations are rare in cancer, understanding the regulation of p63 protein dynamics by post-translational modifications is crucial in targeting the oncogenic activities of ΔNp63. Among modifications, phosphorylation and ubiquitination have impacts on p63 protein stability and transcriptional function, so identification of responsible enzymes and modulation of their activities could be explored as a therapeutic option in tumors with overexpressed ΔNp63. Our findings suggest that the pioneer factor activity of ΔNp63 is intimately linked to TGFβ signaling.

Moreover, a full understanding of the ΔNp63 interactome pattern in tumors may be valuable to enable the development of novel therapeutic approaches, since the abrogation of ΔNp63α interaction with interactors/co-activators could broadly affect ΔNp63-dependent transcription [48]. Our mass spectrometry analysis illustrated a variety of ΔNp63 epigenetic interactors and led to the identification of novel ΔNp63 interactors, NURD and NCOR/SMRT complexes as well as DNMT1, revealing an unexpected effect of TGFβ signaling on the composition of the ΔNp63 interactome. We showed that activation of SMAD proteins by TGFβ stimulation induces dissociation of ΔNp63-NURD and ΔNp63-NCOR/SMRT HDAC3 complexes, whereas it promotes the assembly of a ΔNp63-p300 complex. These observations suggest that activated SMAD2/3 proteins drive the ΔNp63 selectivity for histone modification complexes, significantly affecting the outcome of ΔNp63-dependent transcription. ΔNp63 binds to inaccessible chromatin, showing intrinsic pioneer factor ability. However, since ΔNp63 is bound to NURD and NCOR/SMRT complexes in the absence of TGFβ signals, its presence is not sufficient to induce gene transcription, at least for the genes investigated in the current study. Therefore, an activation of the TGFβ pathway signals the switch of ΔNp63 interactors and opens the ΔNp63-bound chromatin regions, leading to active transcription of genes.

**Fig. 7:**
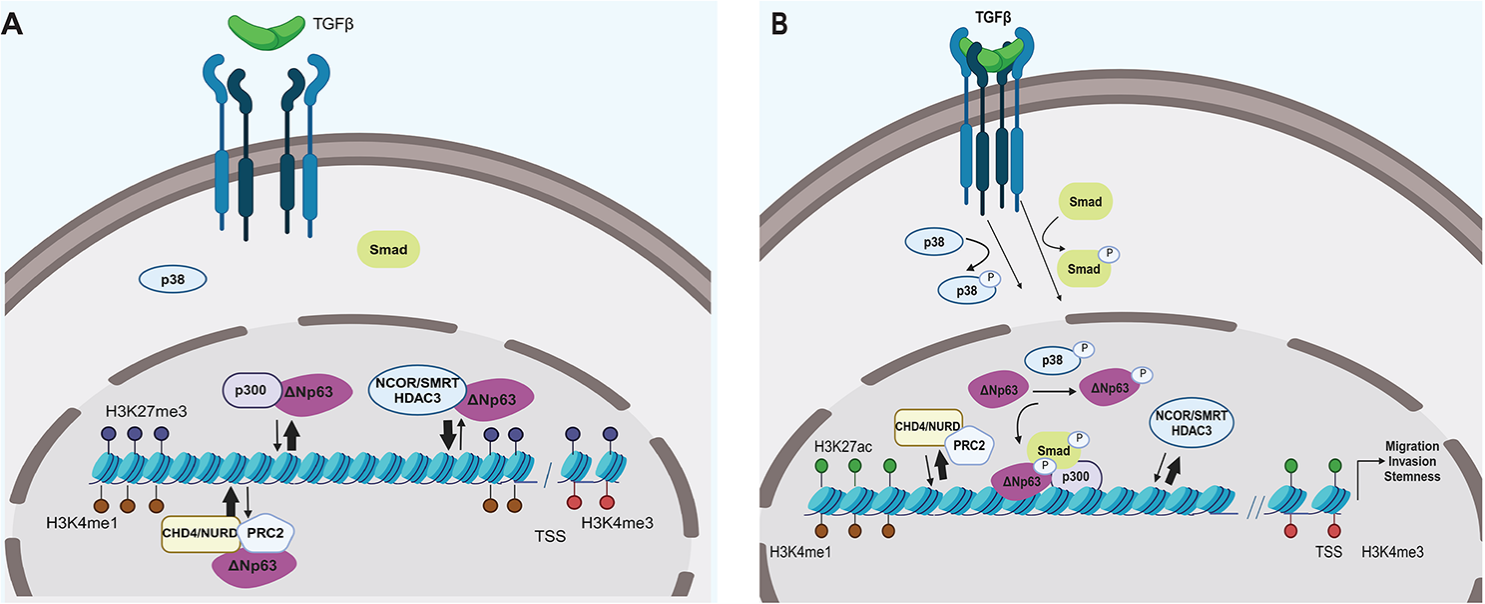
Schematic illustration of the effect of active TGFβ signaling on ΔNp63-dependent transcription. **(A)** In the absence of active TGFβ pathway, ΔNp63 is bound to NURD/PRC2 and NCOR/SMRT/HDAC3 complexes on TGFβ/SMAD target regulatory genomic loci. These regions showing high H3K4me1 are bookmarked for transcription by ΔNp63; however, the presence of the H3K27 tri-methylation mark results in condensed chromatin and inactive transcription. **(B)** Activation of TGFβ signaling leads to phosphorylation of ΔNp63 at Ser66/68 via p38 MAPK, nuclear translocation of SMAD2/3 transcription factors and complex formation between ΔNp63, SMAD2/3 and p300. p300 catalyzes the acetylation of K27 on H3, which promotes chromatin accessibility and activation of gene transcription favoring cancer cell stemness and invasiveness. Dynamic interactions with chromatin are shown with two anti-parallel arrows. Arrow thickness correlates to the prevalent interaction (association or dissociation). Created with BioRender.com

We propose that ΔNp63 bookmarks the TGFβ/SMAD regulatory genomic regions and is essential for the transcriptional regulation of the downstream target genes regulating the sphere forming capacity of breast cancer cells. The ALK5/p38 axis of TGFβ signaling phosphorylates and stabilizes the ΔNp63 protein, whereas activation of the SMAD2/3 axis induces the formation of the ΔNp63-p300 complex, leading to H3K27 acetylation and activation of transcription, promoting cancer cell stemness and invasiveness (Fig. 7).

## Conclusions

We revealed two novel mechanisms of ΔNp63 regulation induced by TGFβ signaling. The first involves the TGFβ/p38 MAPK-dependent phosphorylation of ΔNp63, and the second is the TGFβ/SMAD-induced switching of ΔNp63 epigenetic regulators. These new TGFβ/ΔNp63 links are of high importance for untangling the complexity of ΔNp63 function and understanding the pleiotropic ΔNp63 transcriptional effects, enabling the design of ΔNp63-targeted therapies for cancer and developmental syndromes.

### Electronic supplementary material

Below is the link to the electronic supplementary material.

Supplementary Material 1

Supplementary Material 2

Supplementary Material 3

## Data Availability

No datasets were generated or analysed during the current study.

## Abbreviations

ChIP: Chromatin immunoprecipitation
JNK: c-Jun N-terminal kinase
MAPK: Mitogen-activated protein kinase
NCOR: Nuclear Corepressor
NURD: Nucleosome Remodeling and Deacetylase
TGFβ: Transforming growth factor β
TSS: Transcription start site
