# Supplementary material for: ΔNp63 bookmarks and creates an accessible epigenetic environment for TGFβ-induced cancer cell stemness and invasiveness
Source: Cell Commun Signal. 2024 Aug 23;22:411. doi: 10.1186/s12964-024-01794-5 (PMC11342681; doi:10.1186/s12964-024-01794-5)
Supplement: Supplementary file 2 — Supplementary Material 2 [file 12964_2024_1794_MOESM2_ESM.docx]

**ΔNp63 bookmarks and creates an accessible epigenetic environment for TGFβ-induced cancer cell stemness and invasiveness**

Eleftheria Vasilaki^1,^*, Yu Bai^1^, Mohamad Moustafa Ali^1^, Anders Sundqvist^1,2^, Aristidis Moustakas^1^ and Carl-Henrik Heldin^1,^*

**Additional file 1**

**Table S1.** **Primer sequences used for ChIP-qPCR.** Primer sequences used for ChIP-qPCR are shown. FW, forward primer; Rev, reversed primer

| **Primer sequences for ChIP-qPCR** | |
| --- | --- |
| **Name** | **Sequence** |
| ***LAMB3* FW** | 5'-TTGCCCTGCACTACAACACA-3' |
| ***LAMB3* Rev** | 5'-GTAACACACCAGGCCCACTT-3' |
| ***ITGA2* FW** | 5´-GTCCTGCCTCCCAAACACAGGT-3´ |
| ***ITGA2* Rev** | 5´-CCGGTGACACGATTTTGACGCT-3´ |
| ***SERPINE1* FW** | 5’-GCAGGACATCCGGGAGAGA-3’ |
| **SERPINE1 Rev** | 5’- CCAATAGCCTTGGCCTGAGA-3’ |

**Table S2.** **Primer sequences used for RT-qPCR.** Primer sequences used for RT-qPCR are shown. FW, forward primer; Rev, reversed primer

| **Primer sequences for qRT-PCR** | |
| --- | --- |
| **Name** | **Sequence** |
| ***GAPDH* FW** | 5'-GGAGTCAACGGATTTGGTCGTA-3' |
| ***GAPDH* Rev** | 5'-GGCAACAATATCCACTTTACCA-3' |
| ***ITGA2* FW** | 5'-GTCGGTGCTCCTCGGGCAAA-3' |
| ***ITGA2* Rev** | 5'-TGGTCACCTCGGTGAGCCTGA-3' |
| ***LAMB3* FW** | 5'-ACGGCAGAACACACAGCAAGGA-3' |
| ***LAMB3* Rev** | 5'-ACCGGGTCCTCCCAACAAGCA-3' |
| ***SERPINE1* FW** | 5'-GAGACAGGCAGCTCGGATTC-3' |
| ***SERPINE1* Rev** | 5'-GGCCTCCCAAAGTGCATTAC-3' |
| ***p63 (total) FW*** | 5'-AACCAGAGATGGGCAAGTCCTGGGC-3' |
| ***p63 (total) Rev*** | 5'-ATCCGCCTTCCTGTCTCTTCCTGGG-3' |
| ***ΔNp63 FW*** | 5'-GCCAGAAGAAAGGACAGCAGCA-3' |
| ***ΔNp63 Rev*** | 5'-AGGTTCGTGTACTGTGGCTCACT-3' |
| ***TAp63 FW*** | 5'-CATCCAGCGTTTCGTAGAAACCCCAGC-3' |
| ***TAp63 Rev*** | 5'-GTGTGCTCTGGGACATGGTGGATCG-3' |
