# Supplementary material for: ΔNp63 bookmarks and creates an accessible epigenetic environment for TGFβ-induced cancer cell stemness and invasiveness
Source: Cell Commun Signal. 2024 Aug 23;22:411. doi: 10.1186/s12964-024-01794-5 (PMC11342681; doi:10.1186/s12964-024-01794-5)
Supplement: Supplementary file 3 — Supplementary Material 3 [file 12964_2024_1794_MOESM3_ESM.docx]

**ΔNp63 bookmarks and creates an accessible epigenetic environment for TGFβ-induced cancer cell stemness and invasiveness**

Eleftheria Vasilaki^1,2^*, Yu Bai^1­ ­^, Mohamad Moustafa Ali^1^, Anders Sundqvist^1,3^, Aristidis Moustakas^1^ and Carl-Henrik Heldin^1,^*

**Additional file 3**

**Additional figures and legends**


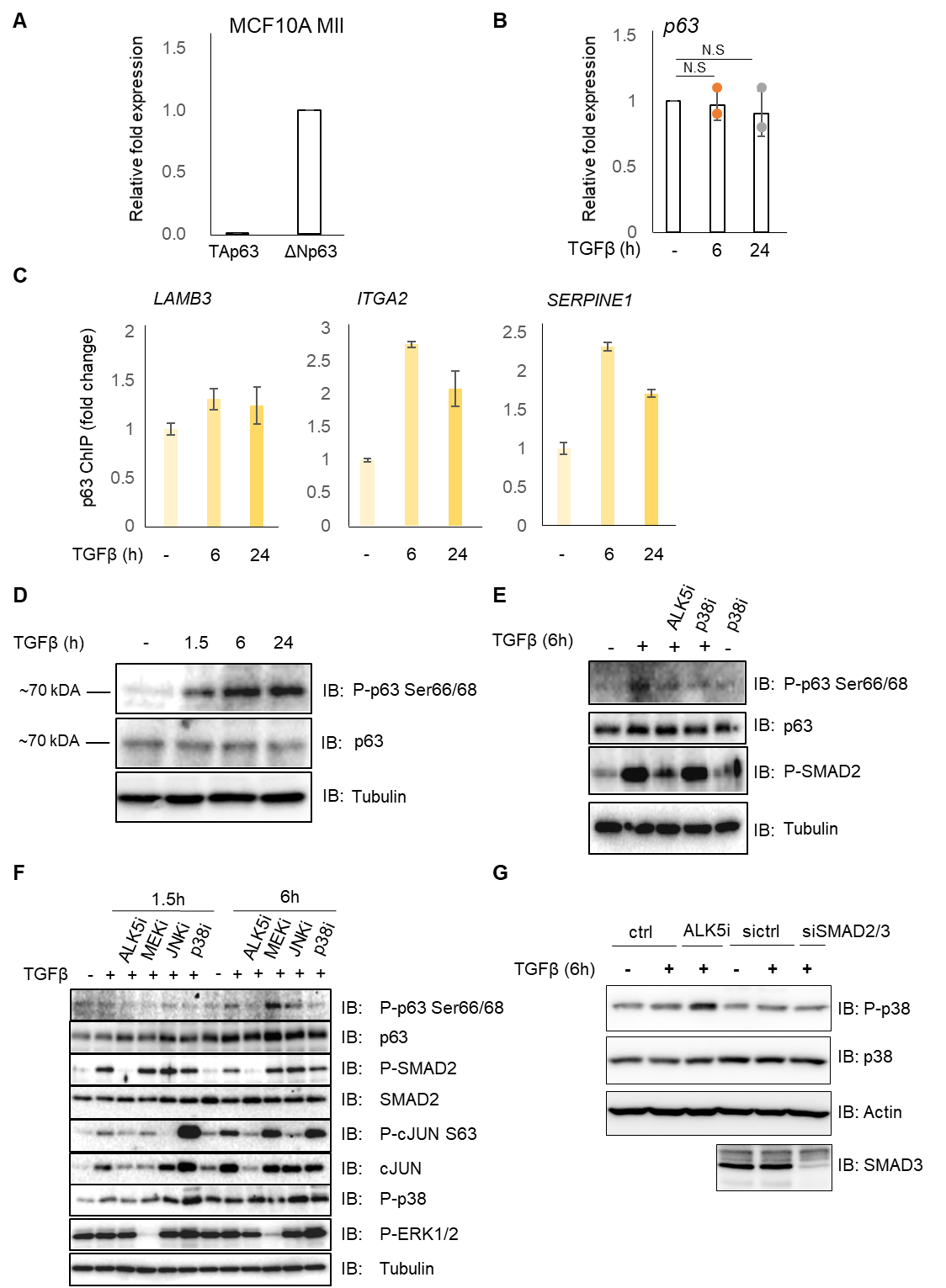


**Supplemental Figure 1 related to Main Figure 1**

**Fig. S1 related to Fig. 1.** Effect of TGFβ stimulation on p63 mRNA expression and recruitment to DNA**. (A, B)** qRT-PCR analysis showing the relative expression of the TA and ΔN isoforms of p63 in MCF10A MII cells (A)**,** as well as the effect of TGFβ stimulation on p63 (total) mRNA expression in these cells (B). Results of three independent experiments are shown as mean ± SD; N.S: not significant difference. **(C)** ChIP-qPCR showing the effect of TGFβ treatment on p63 binding to the indicated gene loci in HCC1954 cells. **(D)** TGFβ stimulation induced phosphorylation of ΔNp63 at Ser66/Ser68. HCC1954 cells were incubated for 24 h in 3% FBS medium before addition or not of TGFβ (5 ng/ml) for the indicated time-periods. Cell lysates were analyzed by IB with the indicated antibodies. **(E, F)** IB analysis of MCF10A MII cells treated or not with indicated kinase inhibitors or DMSO (ctrl) in the presence of TGFβ for 1.5 or 6 h, as indicated. **(G)** IB analysis showing the effect of ALK5 inhibition or SMAD2/3 depletion on p38 activation**.** MCF10A MII cells treated or not with the ALK5 inhibitor, SB505124, or DMSO (ctrl), or transfected with non-targeting control (sictrl) siRNA or with siRNAs specific against SMAD2 and SMAD3 were stimulated with TGFβ for 6 h. Cell lysates were subjected to IB analysis with the indicated antibodies.

**
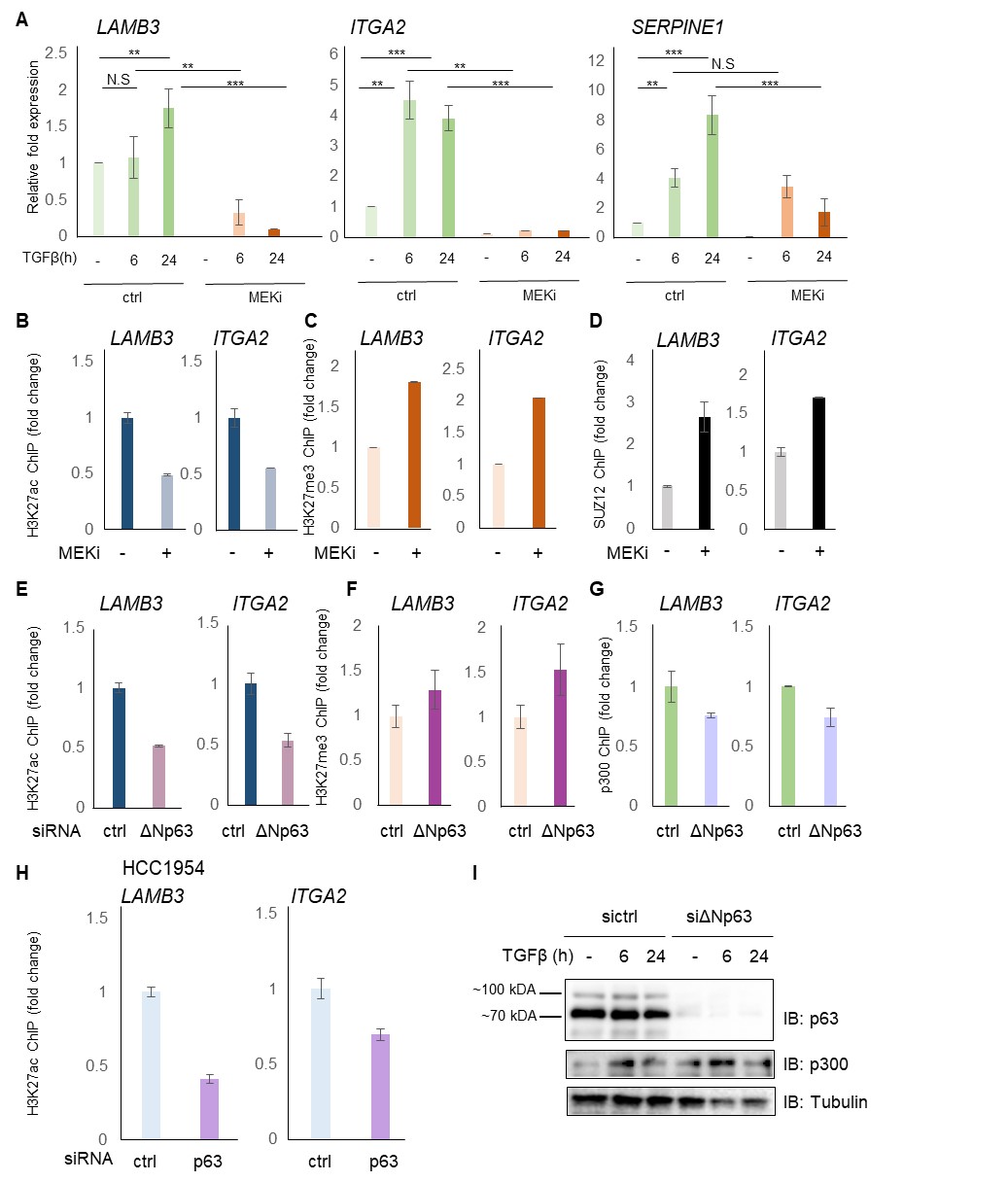
**

**Supplemental Figure 2 related to Main Figure 2**

**Fig. S2 related to Fig. 2.** TGFβ- and EGF-induced gene expression correlates with changes in histone marks orchestrated by p63. **(A)** qRT-PCR analysis of the effect of TGFβ stimulation and MEK inhibition on the expression of *LAMB3*, *ITGA2* and *SERPINE1* genes. MCF10A MII cells were incubated overnight in 0.2% FBS medium before addition of the MEK1/2 inhibitor AZD6244 (MEKi) or DMSO (ctrl). TGFβ was added 20 min later and incubation was prolonged for 6 or 24 h, as indicated. Results of three independent experiments are shown as mean ± SD; * *P* < 0.05, ** *P* < 0.01, *** *P* < 0.001, N.S: not significant difference. **(B, C)** ChIP-qPCR showing the changes in H3K27ac (B) and H3K27me3 (C) histone marks of the indicated gene loci in MCF10A MII cells treated or not with MEK1/2 inhibitor for 6 h. **(D)** ChIP-qPCR showing the effect of MEK inhibition on the recruitment of SUZ12 to the indicated gene loci. MCF10A MII cells treated or not with MEK inhibitor for 6 h and subjected to ChIP with SUZ12 antibody. **(E-G)** ChIP-qPCR showing the effect of ΔNp63 depletion on H3K27ac (E) and H3K27me3 (F) marks as well as p300 recruitment to DNA (G). MCF10A MII cells transfected with non-targeting control (sictrl) siRNA or with siRNAs specific against the ΔN isoforms of p63 were subjected to ChIP with H3K27ac (E), H3K27me3 (F) or p300 antibodies (G) and subsequent qPCR analysis. **(H)** ChIP-qPCR experiment showing the effect of p63 depletion on H3K27ac in HCC1954 cells. **(I)** Effect of ΔNp63 depletion on p300 expression. MCF10A MII cells transfected with non-targeting control (sictrl) siRNA or with siRNA specific against the ΔN isoforms of p63 were treated or not with TGFβ for 6 or 24 h and subjected to immunoblot analysis with the indicated antibodies.

**
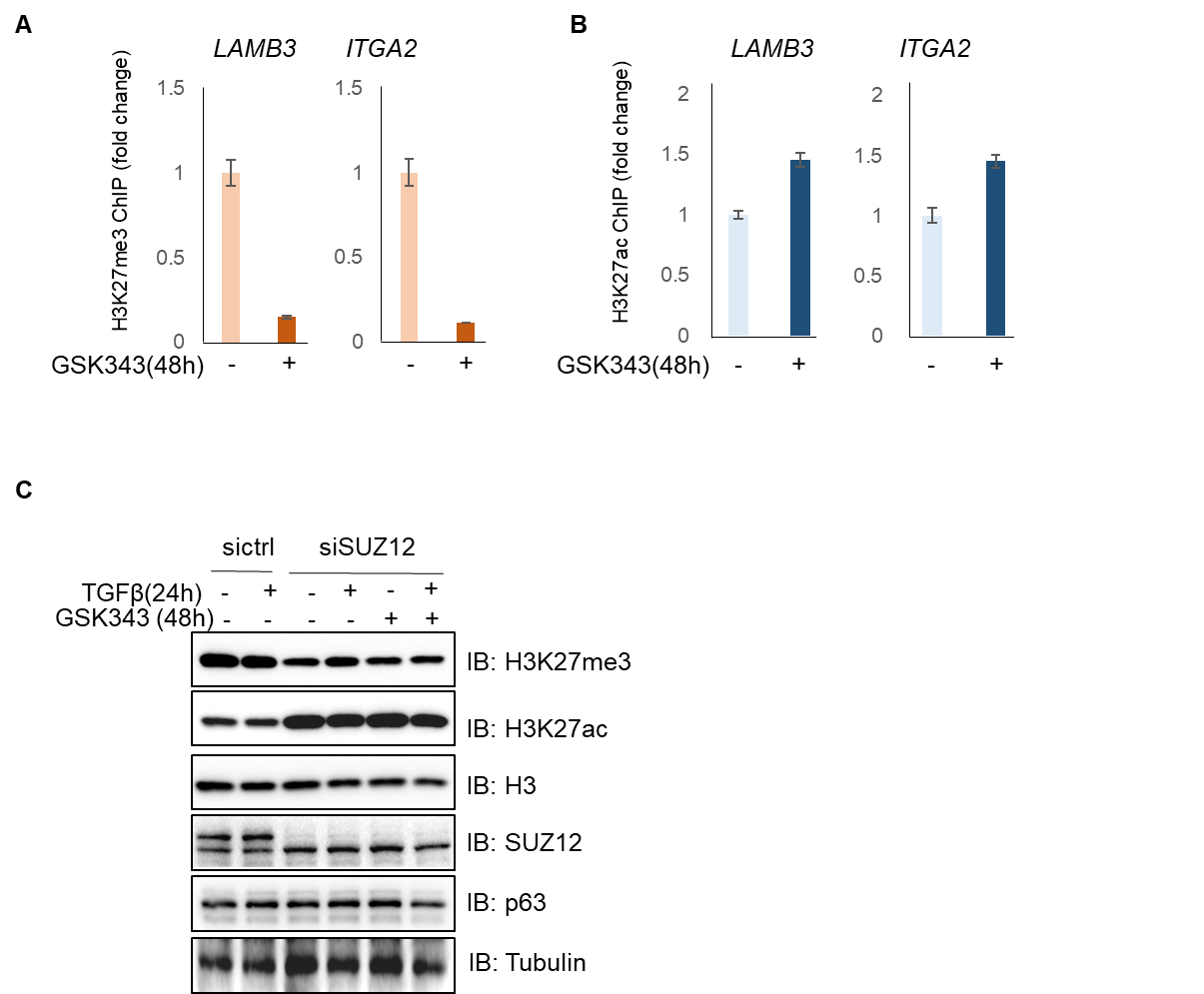
**

**Supplemental Figure 3 related to Main Figure 3**

**Fig. S3 related to Fig. 3.** GSK343 inhibitor or depletion of SUZ12 significantly decreases H3K27me3 and increases H3K27ac. **(A, B)** ChIP-qPCR showing the changes in H3K27me3 (A) and H3K27ac (B) histone modification marks of the indicated gene loci in MCF10A MII cells treated or not with an EZH2 inhibitor (GSK343) for 48 h. **(C)** Effect of GSK343 inhibitor or SUZ12 depletion on total H3K27me3 and H3K27ac levels. MCF10A MII cells transfected with non-targeting control (sictrl) siRNA or with siRNA specific against SUZ12, treated or not with a GSK343 inhibitor for 48 h and stimulated or not with TGFβ for 24 h, were subjected to IB analysis with the indicated antibodies.


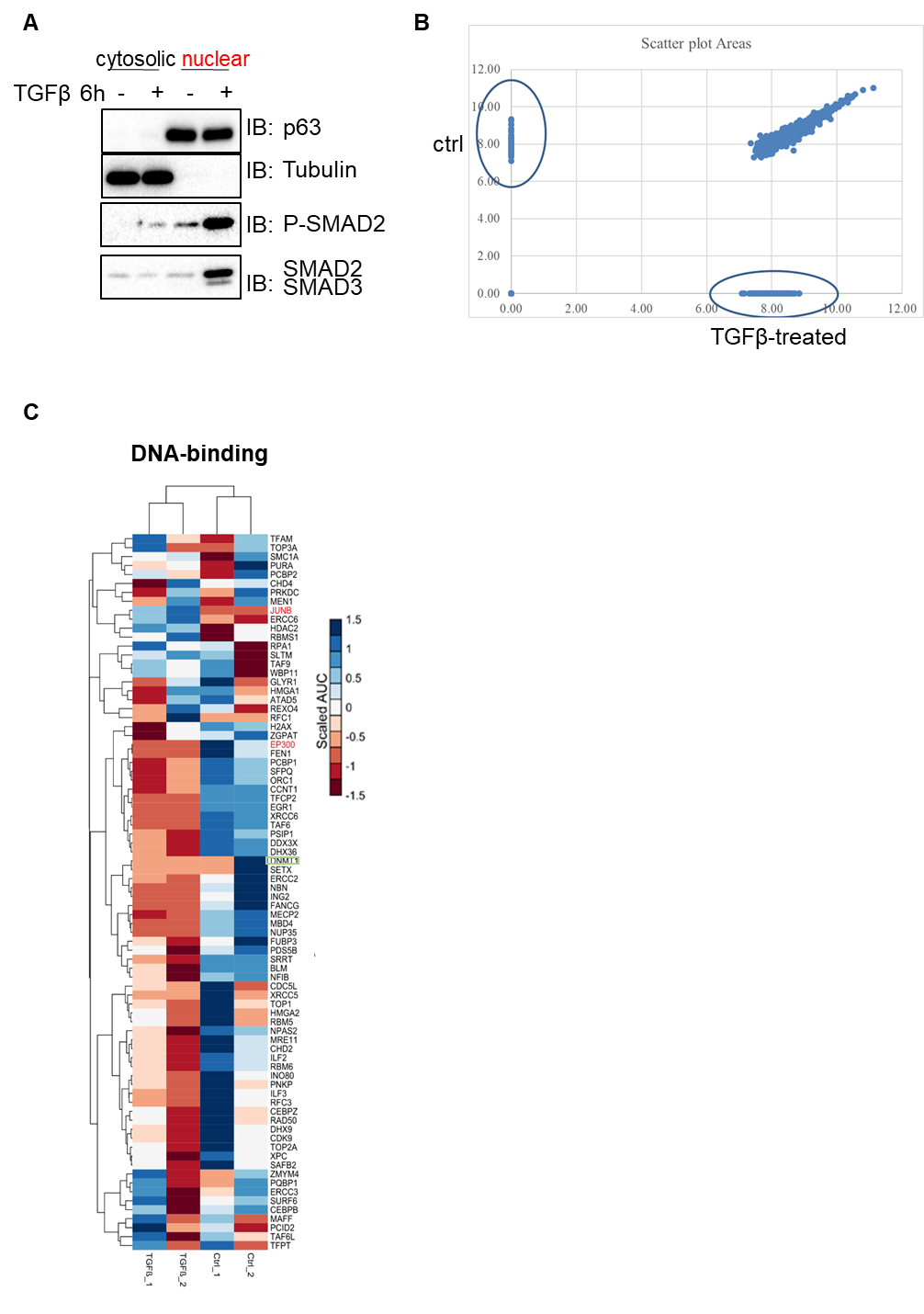


**Supplemental Figure 4 related to Main Figure 4**

**Fig. S4 related to Fig. 4.** Identification of ΔNp63 interactors. **(A)** Preparation of samples for mass spectrometry analysis. MCF10A MII cells, starved in 0.2% FBS medium and stimulated with TGFβ or not for 6 h, were subjected to nuclear-cytosolic fractionation. Cytosolic and nuclear lysates were subjected to IB with the indicated antibodies. **(B)** Scatter plot illustrating the intensities of detected ΔNp63 interactors in the presence (TGFβ–treated) or not (ctrl) of TGFβ for 6 h. **(C)** Heatmap showing the intensity patterns of proteins involved in DNA binding in the respective samples. Scaled AUC expression was calculated using Z-score method representing quantified AUC of peptide intensities.


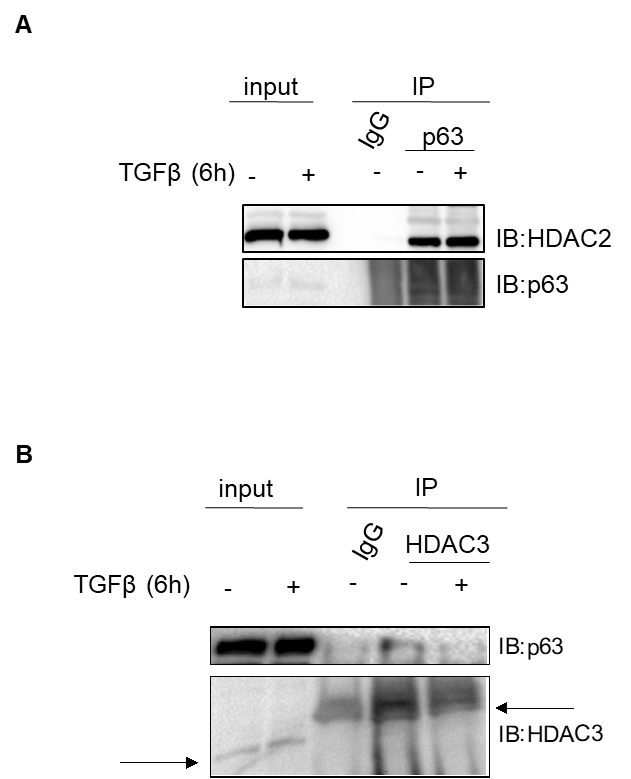


**Supplemental Figure 5 related to Main Figure 5**

**Fig. S5 related to Fig. 5.** ΔNp63 interaction with histone remodeling complexes. **(A, B)** p63 interaction with HDAC2 and HDAC3. MCF10A MII cells, starved in 0.2% FBS medium and stimulated with TGFβ or not for 6 h, were subjected to immunoprecipitation (IP) with a p63-specific antibody (A) or an HDAC3-specific antibody (B), or IgG control, and analyzed by IB with the indicated antibodies. In B, arrows indicate the band detecting HDAC3 expression.

**
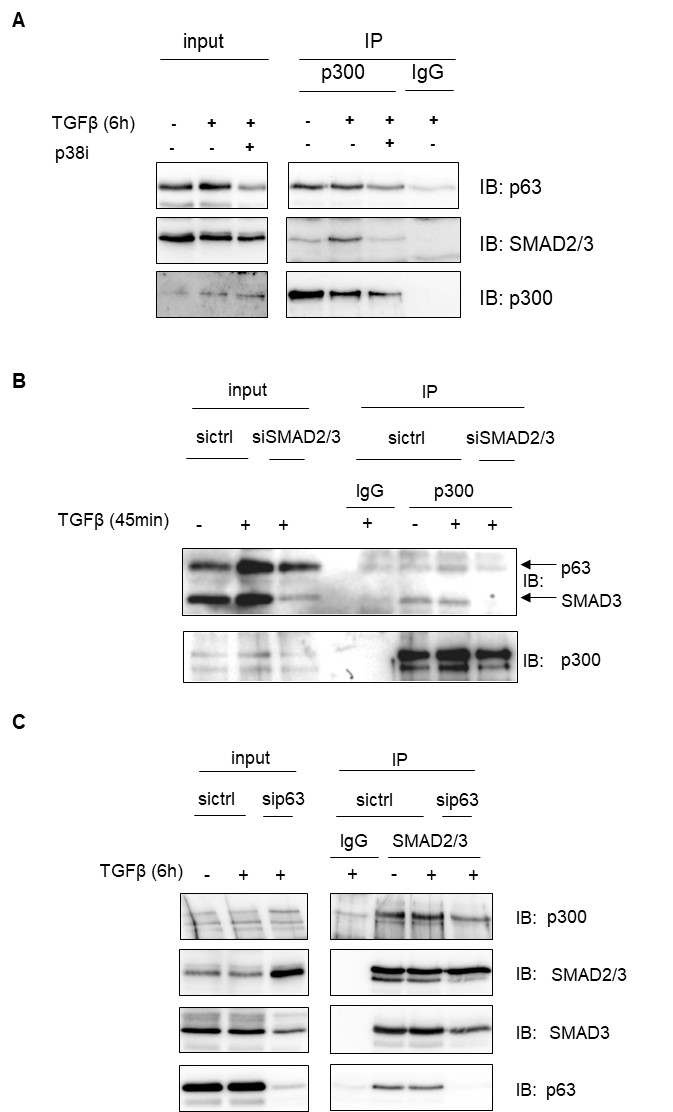
**

**Supplemental Figure 6 related to Main Figure 6**

**Fig. S6 related to Fig. 6.** p63**/**SMAD/p300 complex formation. **(A)** MCF10A MII cells were treated or not with the p38 kinase inhibitor, SB203580, or DMSO (ctrl) in the presence of TGFβ 6 h. Cell lysates were subjected to immunoprecipitation (IP) with a p300-specific antibody or IgG control, and analyzed by immunoblotting (IB) with the indicated antibodies. **(B)** MCF10A MII cells transfected with non-targeting control (sictrl) siRNA or with siRNAs specific against SMAD2 and SMAD3 were incubated in 0.2% FBS medium overnight and treated or not with TGFβ for 45 min. Cell lysates were subjected to IP with a p300-specific antibody or IgG control, and analyzed by IB with the indicated antibodies. **(C)** MCF10A MII cells transfected with non-targeting control (sictrl) siRNA or with siRNA specific against all p63 isoforms were incubated in 0.2% FBS medium overnight and treated or not with TGFβ for 6 h. Cell lysates were subjected to immunoprecipitation (IP) with a SMAD2/3-specific antibody or IgG control, and analyzed by immunoblotting with the indicated antibodies.
